# Insights Into Sexual Maturation and Reproduction in the Norway Lobster (*Nephrops norvegicus*) via *in silico* Prediction and Characterization of Neuropeptides and G Protein-coupled Receptors

**DOI:** 10.3389/fendo.2018.00430

**Published:** 2018-07-27

**Authors:** Tuan V. Nguyen, Guiomar E. Rotllant, Scott F. Cummins, Abigail Elizur, Tomer Ventura

**Affiliations:** ^1^GeneCology Research Centre, Faculty of Science, Health, Education and Engineering, University of the Sunshine Coast, Sunshine Coast, QLD, Australia; ^2^Institute de Ciències del Mar, Consejo Superior de Investigaciones Científicas, Passeig Marítim de la Barceloneta, Barcelona, Spain

**Keywords:** crustacea, data mining, neurohormone, neuropeptides, neuropeptidome, GPCRs, phoenixin

## Abstract

Multiple biological processes across development and reproduction are modulated by neuropeptides that are predominantly produced and secreted from an animal's central nervous system. In the past few years, advancement of next-generation sequencing technologies has enabled large-scale prediction of putative neuropeptide genes in multiple non-model species, including commercially important decapod crustaceans. In contrast, knowledge of the G protein-coupled receptors (GPCRs), through which neuropeptides act on target cells, is still very limited. In the current study, we have used *in silico* transcriptome analysis to elucidate genes encoding neuropeptides and GPCRs in the Norway lobster (*Nephrops norvegicus*), which is one of the most valuable crustaceans in Europe. Fifty-seven neuropeptide precursor-encoding transcripts were detected, including phoenixin, a vertebrate neurohormone that has not been detected in any invertebrate species prior to this study. Neuropeptide gene expression analysis of immature and mature female *N. norvegicus*, revealed that some reproduction-related neuropeptides are almost exclusively expressed in immature females. In addition, a total of 223 GPCR-encoding transcripts were identified, of which 116 encode GPCR-A (Rhodopsin), 44 encode GPCR-B (Secretin) and 63 encode other GPCRs. Our findings increase the molecular toolbox of neural signaling components in *N. norvegicus*, allowing for further advances in the fisheries/larvae culture of this species.

## Introduction

The Norway lobster (*Nephrops norvegicu*s) is widely distributed in the North-Eastern Atlantic Ocean and parts of the Mediterranean Sea, where it is economically important for many countries in the area (1). Harvest of *N. norvegicus* has steadily increased since the 1950s from ~10,000 tons per year, to more than 70,000 tons in the 2000s, yet has decreased to ~50,000 tons per year from 2010 (FAO 2015). Potential improper fishing strategies (leading to probable overexploitation) have been indicated as the main reason for this decline (2, 3), while diseases, climate change and sea pollution (e.g., microplastics, heavy metal contamination, and endocrine disruptors) have also been implicated (4, 5). To address these issues, there is a growing interest in adopting hatchery technologies, aquaculture and restocking for *N. norvegicus* (e.g., (6); Project NEPHROPS, http://cordis.europa.eu/project/rcn/103402_en.html). However, these practices are still in their infancy due to a number of limiting factors, including low fecundity, fragile larvae, and cannibalism of post larvae and juveniles (5). To better enable these technologies, an in-depth knowledge of the species' reproductive biology is critical.

Adult *N. norvegicus* are estimated to reach maturity at around 2–3 years (7). A number of reproduction-related studies have been performed, including the thorough investigation of its ovarian cycle (8–14). *N. norvegicus* undergoes cyclic ovarian maturation that consists of 4 primary stages based on the ovary anatomy (12). Physiological processes rely on multiple external (e.g., temperature, pheromones) and internal cues. Among the internal cues are neurohormones including neuropeptides, which are paramount for numerous complex neuroendocrine processes. Upon synthesis and release from neural cells, neurohormones bind to receptors present on target cells and initiate multiple downstream cascades (15). Neuropeptides are derived from precursor neuropeptides, which contain a signal peptide and often sites for post-translational processing, such as proteolytic cleavage, N-terminal pyrolation, C-terminal amidation, and disulphide bonding (15).

Over the past few decades, a number of key neuropeptides have been identified in crustaceans that have functions related to sexual maturation and reproduction (16–18). For example, the gonad inhibiting hormone/vitellogenesis inhibiting hormone (GIH/VIH), is a member of the crustacean hyperglycemic hormone (CHH) family, which includes additional members like ion transport peptide (ITP) and molt-inhibiting hormone (MIH). Together, the CHH family is a hallmark of crustacean development and reproduction and has been studied intensively [Refer to an comprehensive review by Webster et al. (19). GIH/VIHs are produced and secreted predominantly by the X-organ-sinus gland neuroendocrine center (XO-SG) in the eyestalk and act to inhibit gonad development. Also, several studies propose an unidentified gonad stimulating hormone (GSH) acting downstream of GIH that is secreted from the brain and/or thoracic ganglia (20, 21). In an attempt to identify a GSH in crustaceans, several studies using multiple species, have described a gonadotropin-releasing hormone (GnRH)-like peptide (22–25). However, solid evidence that this peptide has a role in reproduction *in vivo* has not yet been established (26).

Another group of peptide hormones that have gained increasing attention with regards to their potential for enhancing crustacean aquaculture practice is the insulin-like peptides (ILPs). Initially, a single ILP was identified in decapods, namely the androgenic gland hormone (IAG) (27). Silencing the gene encoding IAG in the giant freshwater prawn *Macrobrachium rosenbergii* enabled a full functional sex reversal of males into neo-females (28). These neo-females are fertile and able to breed with males, which results in the production of all-male populations, an advantageous goal of large scale aquaculture (29). This hormone, which controls masculinity in crustaceans, cannot be considered a classical neuropeptide since its expression is exclusive to a male-specific endocrine gland, namely the androgenic gland. Recent studies identified non-IAG ILPs in the eastern spiny lobster *Sagmariasus verreauxi* (30) as well as the Australian redclaw crayfish *Cherax quadricarinatus* (31) and were shown to be transcribed within the central nervous system (CNS) and are evolutionarily related to previously identified ILPs in other arthropod groups.

To add to the complexity of clearly defining crustacean neuropeptides, many neuropeptides are pleiotropic and novel functions are being described for previously characterized neuropeptides. Recent studies have found that several neuropeptides are now also involved in crustacean reproduction, including the red pigment concentrating hormone (RPCH) (32), neuroparsin (33), neuropeptide F (34), and pigment-dispersing hormone (PDH) (35). Moreover, the roles of the vast majority of neuropeptides identified in crustaceans have not been clarified yet, highlighting the gap in crustacean neuropeptide research, including those related to reproduction and sexual maturation.

Neuropeptides predominantly bind and activate cell surface G protein-coupled receptors [GPCRs; with few exceptions like the tyrosine kinase insulin receptors (36, 37)]. GPCRs are an ancient family of proteins that act as signal transducers that consist of an extracellular N-terminus, a region of seven transmembrane (7-TM) domain and an intracellular C-terminus. Extracellular ligand binding changes the intracellular C-terminus conformation, leading to activation of an associated G-protein, which initiates a signal transduction. Arthropod GPCRs are classified into superfamilies based on sequence motifs including: typical GPCRs (Rhodopsin-like, Secretin-like, metabotropic glutamate–like receptors) and atypical GPCRs (Frizzled, Bride of sevenless, chemokine receptors, etc.) (38). It is important to note that GPCRs from related species usually do not share high overall sequence homology as neuropeptides do, therefore *in silico* deorphanizing GPCRs is considered as a very difficult task (39).

With the recent advancement of Next Generation Sequencing (NGS), even with the lack of a sequenced genome, it is now possible to use bioinformatic approaches to identify neuropeptides and their predicted GPCRs using transcriptomic databases. *In silico* neuropeptide mining of transcriptomes have been applied in a variety of crustaceans, including crabs (40–42), prawns (43), crayfishes (44, 45), and lobsters (46, 47). Fewer NGS-based studies provided insights into GPCRs in decapod crustaceans (47–49). This approach for identification of neuropeptides and GPCRs across decapods facilitates comparative analysis and provides useful targets for functional analysis and attempts to manipulate animal physiology.

This study presents the most comprehensive curation of transcripts encoding putative neuropeptides and GPCRs in *N. norvegicus*, highlighting several reproduction-related neuropeptides. Given the commercial importance of *N. norvegicus*, a deeper understanding of the neurohormonal signaling components, especially those related to reproduction and sexual maturation, might be useful for re-stocking and supporting fisheries to meet the growing demand.

## Materials and methods

### Neuropeptide prediction

Neuropeptides were predicted based on an *N. norvegicus* multi-tissue transcriptome *de novo* assembly performed in a previous study (50). This reference transcriptome was generated using brain, thoracic ganglia, eyestalk, gonad and hepatopancreas samples of *N. norgevicus* females (mature and immature) and males (immature). The final reference transcriptome was scanned against the NCBI non-redundant (nr) database for annotation of transcripts using BLAST+ (51). Annotated sequences were scanned for previously known neuropeptides, or known conserved amino acid motifs such as “FLGFamide,” based on previously highlighted motifs (15). Putative neuropeptide transcript sequences were converted to amino acids (aa) using the Expasy translate tool (http://web.expasy.org/translate/). These sequences were then re-validated using BLASTP. Confirmation of neuropeptide sequences was done using a previously described method (45) with SignalP 4.0 (52), TargetP 1.1 (53) and NeuroPred (54). Neuropeptide aa sequences were saved as FASTA files for ease of analysis. Schematics of neuropeptide precursors were illustrated using the Illustrator for Biological Sequences (IBS) software v1.1 (55).

### Mining of phoenixin ortholog in other species

After the identification of phoenixin (PNX), to identify orthologs of *N. norvegicus* PNX in multiple decapod crustacean species, the full-length neuropeptide extracted from the *de novo* assembly were subjected for a Tblastn search against the human, mouse, and crustaceans NCBI TSA (Transcriptome shotgun assembly) databases and several in-house crustacean transcriptomes. cDNA sequences were converted to aa using the Expasy translate tool (http://web.expasy.org/translate/) and follow the neuropeptide prediction pipeline as described above.

### Tissue distribution of neuropeptide genes

To test the tissue distribution of neuropeptide transcripts, adult *N. norvegicus* were collected offshore from Barcelona harbor (Spain) from the trawling fishing vessel Maireta III. Tissues were dissected on ice and put immediately to RNA-later (Ambion) solution and stored at −80°C until used. Stage of ovarian cycle (stage II–immature, stage IV–mature) was determined based on ovary color scale, gonad somatic index (GSI) and histological organization of the ovaries (12). RNA was extracted from multiple tissues of 4 immature females and 4 mature females. Total RNA from the central nervous system (brain, thoracic ganglia, eyestalk), ovary, hepatopancreas and muscle was extracted using RNAzol®RT (Molecular Research Center Inc., USA) as per manufacturer's recommendation. RNA samples (~1 μg RNA per tissue) were then reverse-transcribed (RT) into cDNA using Tetro cDNA synthesis kit (Bioline, UK). Nine neuropeptide genes and one housekeeping gene were randomly chosen to test the RT-PCR spatial expression. Primers were designed using Primer3 (http://primer3.ut.ee/) based on the CDS of transcripts. A list of primers used can be found in Supplementary Material S1. Reverse transcriptase polymerase cycle reactions (RT-PCRs) were carried out using a touch-down program to allow most products to be amplified with minimal non-specific signal. PCR settings were 94°C for 3 min, followed by 37 cycles of touch-down, 94°C for 30 s, 62–57°C for 30 s (with 1°C decrement for each of the first 6 cycles) and 72°C for 45 s. Following PCR, products were loaded onto an agarose gel (1.5 in 0.6% Tris/Borate/EDTA [TBE] buffer) with ethidium bromide (0.01 μg/mL), electrophoresed for 30 min at 120V, 0.4 mA and documented using a Gel Doc™ XR+ Gel Documentation System (Bio-Rad, CA, USA). The entire gel images of all RT-PCR reactions can be found in Supplementary Material S2.

### GPCR prediction

The previously described *N. norvegicus de novo* assembly (50) was submitted to TransDecoder (http://transdecoder.sf.net) to detect the longest coding regions within the given transcript sequence. The resulting open reading frames (ORFs) were screened against the Pfam database using an implemented plugin in CLC Genomics Workbench v9.5 (https://www.qiagenbioinformatics.com/). An e-value threshold of 1.00 e^−3^ was used in the analysis. All sequences were extracted and stored in a FASTA file as a reference. Predicted structural GPCR domains including 7-TM, and intra/extracellular loops were analyzed using the Pfam-v27 module in CLC Genomics Workbench v9.5. GPCRs were classified into subgroups based on their PFAM annotation. Briefly, GPCRs that are assigned with annotation 7tm_1 (PF00001) were grouped into Rhodopsin group, 7tm_2 (PF00002) were grouped into group, and GPCRs of any other annotation (7tm_3, GPCR_Srx, GPCR_Srw…) were grouped into the “Other” group. Transmembrane domains from putative GPCRs sequences were extracted based on PFAM results. Following multiple sequence alignment, duplicates of identical aa sequences were removed. GPCR sequences with less than 5 transmembrane helices were discarded. All sequences were then combined with a list of previously characterized GPCRs (Supplementary Material S3). List of GPCRs from others arthropods species were adapted from Buckley et al. (48). Global alignments were conducted using ClustalW Thompson et al. (56). Preliminary phylogenies of GPCRs were constructed with the CLC Genomics Workbench v9.5 using maximum likelihood phylogenies estimation based on neighbor-joining initial tree with 1000 bootstraps. JTT protein substitution were used in the analysis (57). For illustration purposes, phylogenetic trees from CLC were imported into the iTOL webserver (58). Two-dimensional (2D) structure of candidate GPCRs were generated using the online visualization web service Protter (http://wlab.ethz.ch/protter/#) with default parameters (Phobius was chosen as default predictor for transmembrane helices prediction).

### *In silico* neuropeptide expression

A FASTA file consisting of nucleotide sequences of all predicted neuropeptides was translated to the coding sequences (CDS) using ORFfinder (https://www.ncbi.nlm.nih.gov/orffinder/). Sequences were manually checked to confirm that the correct coding region was included. Central nervous tissues (brain, eyestalk, and thoracic ganglia), hepatopancreas and female gonads (mature and immature) were chosen to test the expression pattern of neuropeptides. Raw reads from these tissues in immature and mature females were mapped back to the CDS sequence using CLC Genomics Workbench RNA-seq module v9.5 (https://www.qiagenbioinformatics.com/) with the following parameters: Minimum length fraction−0.75, Minimum similar fraction−0.85, other parameters were kept as default. Transcripts were normalized using Fragment per kilobase of transcript per million mapped reads (FPKM). All FPKM values were exported to Microsoft Excel and were color coded based on percentile of distribution.

### Neuropeptides and receptors phylogenetic analysis

After phylogenetic analyses of GPCR-A, GPCR-B, and GPCR-other was generated using CLC Genomics Workbench. Phylogenetic analyses of neuropeptides as well as more refined, shortlisted groups of GPCRs, were generated using multiple sequence alignments with CLUSTALW algorithm (56) imported into MEGA 7.0 (59). Maximum likelihood trees, based on the JTT matrix-based model (57) were conducted with 1,000 bootstraps trials. A list of GnRH superfamily receptors from multiple species was adapted from Hauser and Grimmelikhuijzen (60).

## Results

### *In silico* mining of putative neuropeptides

By employing the described neuropeptide prediction pipeline, we could identify 57 putative neuropeptide precursor transcripts from the *de novo* reference transcriptome of *N. norvegicus*, including most neuropeptides previously identified in other crustacean/insect species (for species comparison, see Supplementary Material S4). A schematic representation of each derived neuropeptide precursor, including sites of bioactive mature peptides, location of cleavage sites as well as precursor size can be viewed in Supplementary Material S5. The aa sequences of all neuropeptide precursors are available in the Supplementary Material S6.

**Figure 1:**
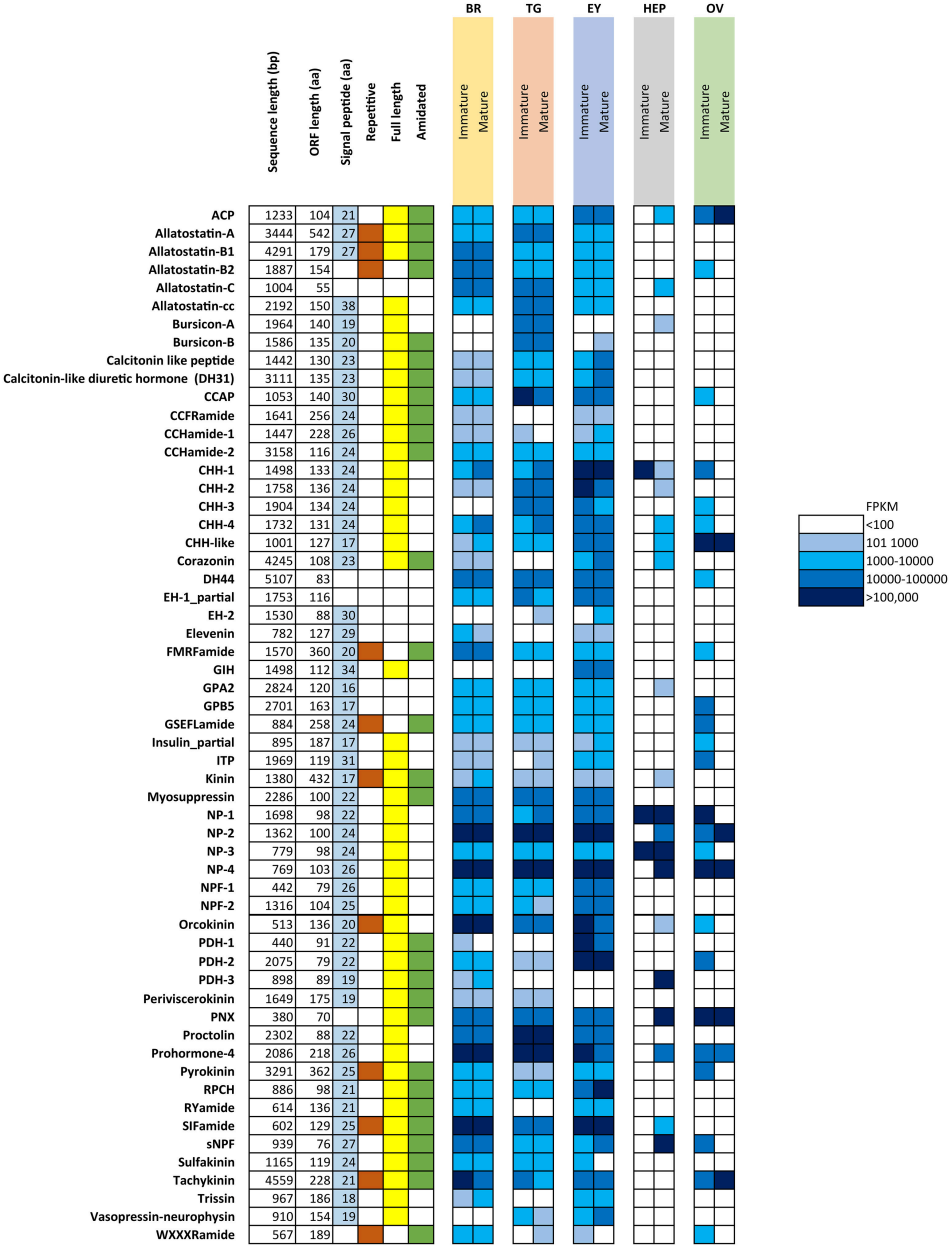
Overview of putative neuropeptides identified in *N. norvegicus*. Shading on signal peptide, repetitive, full length and amidated squares indicates the sequence characteristic identified in each putative neuropeptide. White cells indicate an absence of the sequence character. FPKM in females are color-coded as described in legend. Br, Brain; TG, Thoracic ganglia; EY, Eyestalk; HEP, Hepatopancreas; OV, Ovary. ACP, Adipokinetic hormone/Corazonin-related peptide; CCAP, Crustacean cardioactive peptide; CHH, Crustacean hyperglycemic hormone; DH44, Diuretic hormone 44; EH, Eclosion hormone; GIH, Gonad inhibiting hormone; GPA2, Glycoprotein alpha-2; GPB-5, Glycoprotein beta-5; ITP, Ion transport protein; NP, Neuroparsin; NPF, Neuropeptide F; PDH, Pigment dispersing hormone; PNX, Phoenixin; RPCH, Red pigment concentrating hormone; sNPF, short Neuropeptide F.

Figure 1 summarizes the characteristics of all identified putative neuropeptide precursors, as well as their RNA-seq FPKM expression level for comparison between mature and immature females. Expression analysis showed that the majority of neuropeptides are expressed in the CNS tissues (brain, thoracic ganglia, eyestalk), yet some are exclusive to a single tissue, including the pigment dispersing hormone-3 (brain), gonad inhibiting hormone (eyestalk) and bursicon-A (thoracic ganglia). A clear difference can be noted with a higher abundance of neuropeptide genes expressed in the immature female ovary, when compared with the mature female ovary. The opposite pattern was found in the hepatopancreas, where more neuropeptide genes are expressed in the mature female in comparison with the immature female.

### Tissue distribution of neuropeptides using RT-PCR

**Figure 2:**
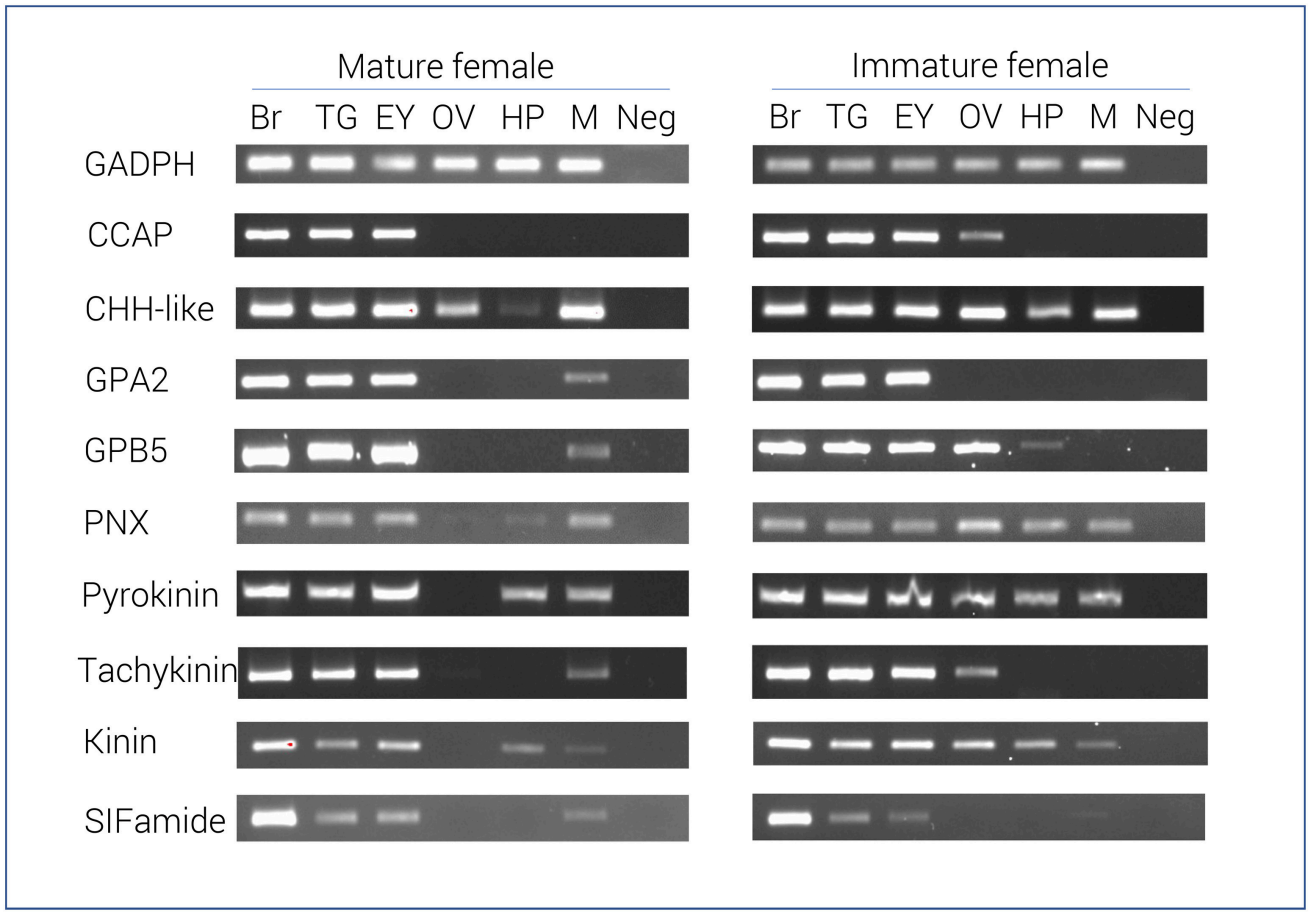
Tissue specific expression of *N. norvegicus* neuropeptide genes using RT-PCR. Spatial expression of neuropeptide genes using gene-specific primers, as well as GADPH gene (control). PCR used cDNA derived from tissues of either mature or immature females. Negative control represents no cDNA in PCR. Br, Brain; TG, Thoracic ganglia; EY, Eyestalk; OV, Ovary; HP, Hepatopancreas; M, Muscle; Neg, Negative control. GADPH, Glyceraldehyde 3-phosphate dehydrogenase; CCAP, Crustacean cardioactive peptide; CHH-like, Crustacean hyperglycemic hormone. GPA2, Glycoprotein alpha-2; GPB5, Glycoprotein beta-5; PNX, Phoenixin.

RT-PCR results of 9 select neuropeptide precursors were illustrated in Figure 2. In general, most neuropeptide amplicons could be detected in CNS tissues (brain, eyestalk, and thoracic ganglia). In support of the RNA-seq results, the majority of neuropeptide transcripts were present in the immature female ovary, yet not detected in the mature ovary. However, a correlation between RNA-seq analysis and RT-PCR in hepatopancreas was not clearly observed. As expected, the reference gene, glyceraldehyde 3-phosphate dehydrogenase (GADPH) was consistently express in all tissues tested and not found in the negative control.

### *In silico* mining of putative GPCRs

Based on the *de novo* reference transcriptome, we could detect 223 putative GPCRs that could be clustered using phylogenetic analyses into 3 groups: a Rhodopsin group (GPCR-A), a Secretin group (GPCR-B), and a group that does not fit into either Rhodopsin or Secretin class, thus termed “other.” A full list of receptors, including receptor isoforms can be found in (Supplementary Material S7).

**Figure 3:**
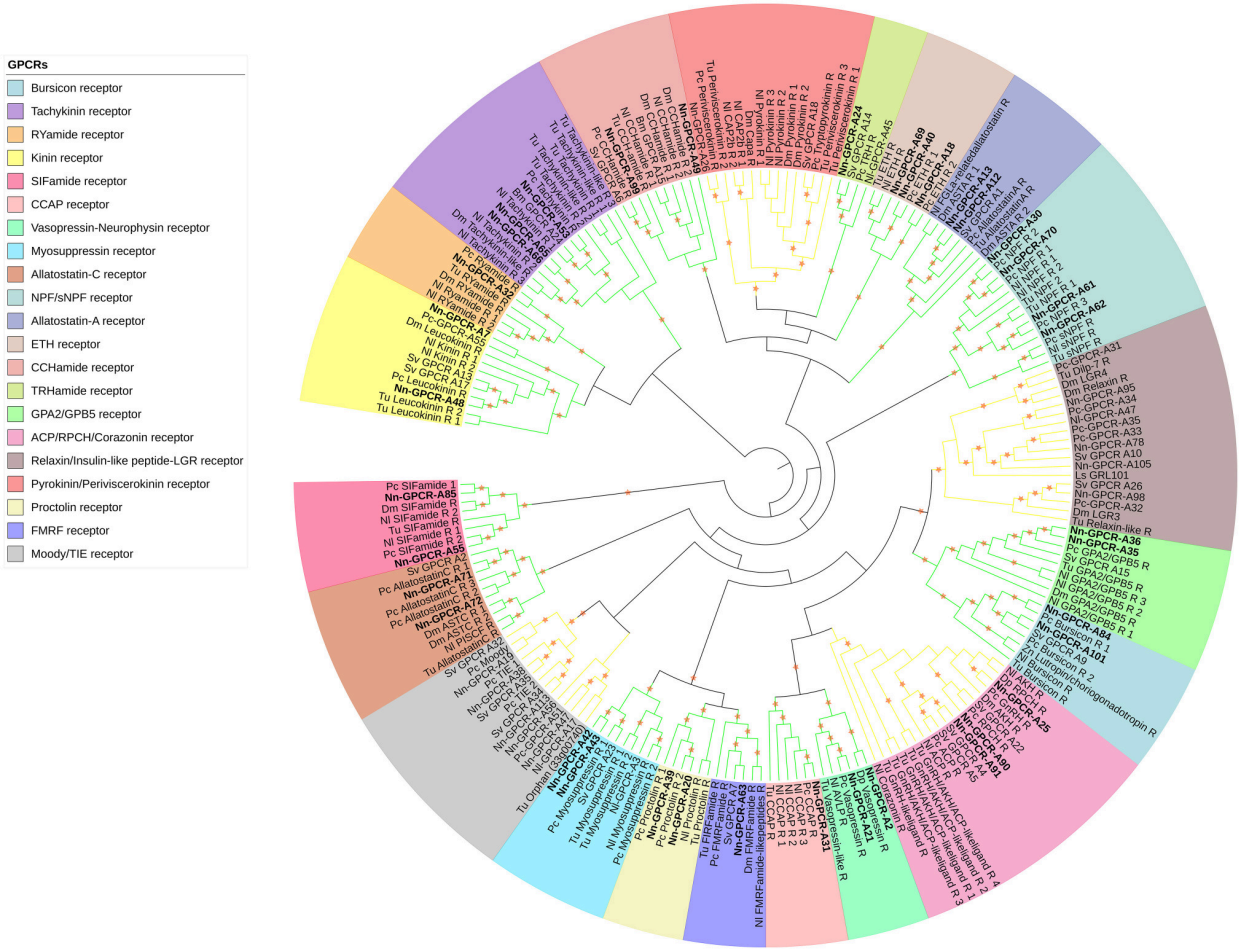
Phylogenetic tree of Rhodopsin class GPCR (GPCR-A) of *N. norvegicus* and other invertebrate species. *N. norvegicus* GPCR clustering with high confidence are highlighted in bold. Orange stars represent clades with a bootstrap value larger than 70. Green line: Clade annotated with high confidence. Yellow line: Clade annotated with low confidence. Red line: Unannotated clade. Bm, *Bombyx mori*; Dp, *Daphnia pulex*; Dm, *Drosophila melanogaster*; Nn, *Nephrops norvegicus*; Nl, *Nilaparvata lugens*; Pc, *Procambarus clarkii*; Sv, *Sagmariasus verreauxi*; Tu, *Tetranychus urticae*; Zn, *Zootermopsis nevadensis*.

**Figure 4:**
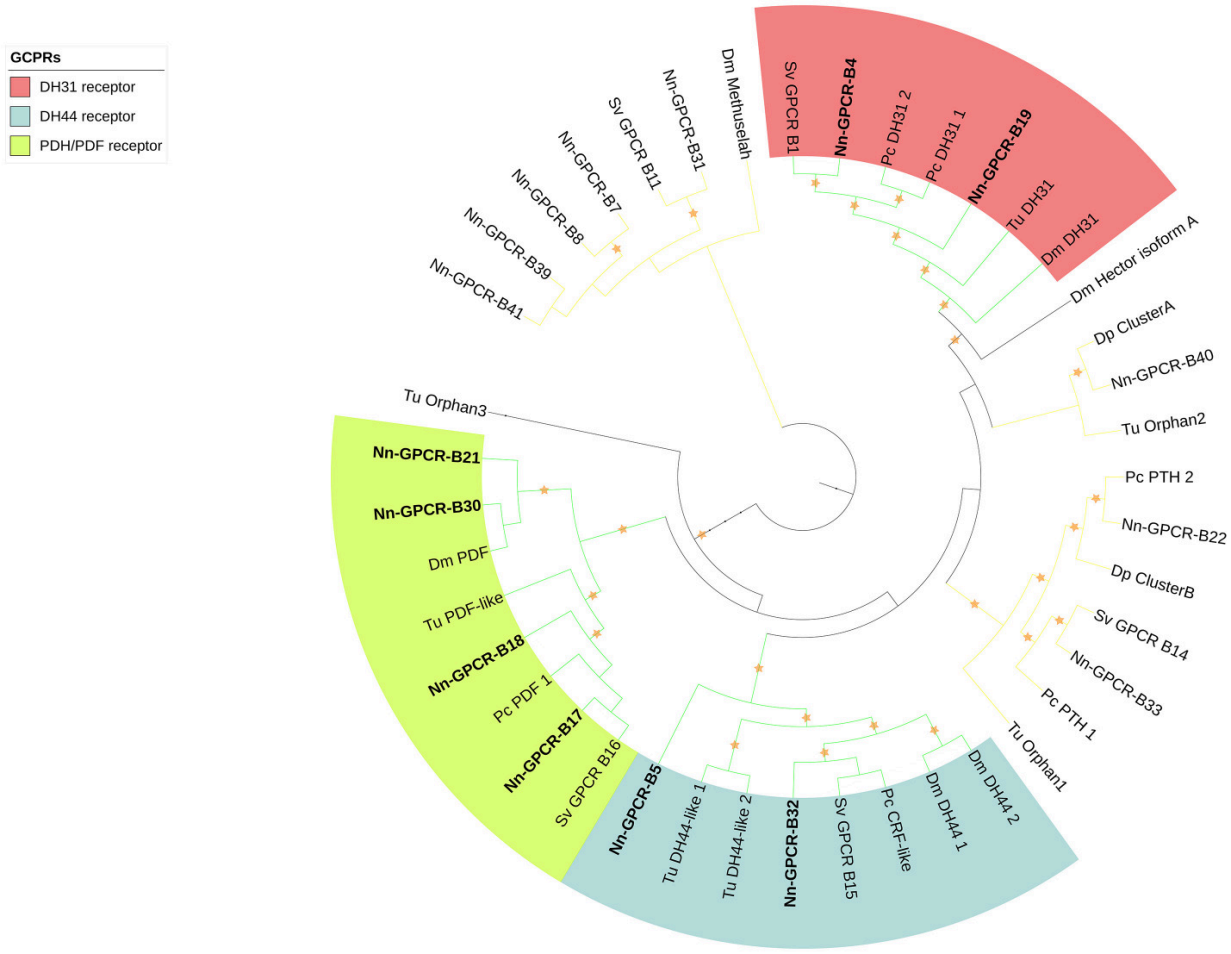
Phylogenetic tree of Secretin class GPCR (GPCR-B) of *N. norvegicus* and other invertebrate species. *N. norvegicus* GPCR(s) clustering with high confidence are highlighted in bold. Orange stars represent clades with bootstrap value larger than 70. Green line: Clade annotated with high confidence. Yellow line: Clade annotated with low confidence. Red line, Unannotated clade. Dm, *Drosophila melanogaster*; Dp, *Daphnia pulex*; Pc, *Procambarus clarkii*; Sv, *Sagmariasus verreauxi*; Tu, *Tetranychus urticae*.

Following phylogenetic analysis, we found that 116 putative GPCRs were GPCR-A (Figure 3) including receptors for FMRFamide, myosuppressin, allatostatin, proctolin, crustacean cardioactive peptide (CCAP), adipokinetic hormone-related neuropeptide/corazonin-related peptide (ACP), red pigment concentrating hormone (RPCH), vasopressin-neurophysin (V-N), CCHamide, thyrotropin-releasing hormone (TRH), Glycoprotein Alpha-2 (GPA2), Glycoprotein Beta-5 (GPB5), Ecdysis-triggering hormone (ETH), SIFamide, kinin, leucokinin, RYamide, sulfakinin, tachykinin, Neuropeptide F (NPF), and short Neuropeptide F (sNPF). Phylogenetic analysis of 44 putative GPCR-B is shown in Figure 4. Comparative phylogenetics resulted in 3 putative GPCR families within GPCR-B with high-confidence value, which include the diuretic hormone 31 (DH31), diuretic hormone 44 (DH44), pigment dispersing hormne (PDH) receptors.

**Figure 5:**
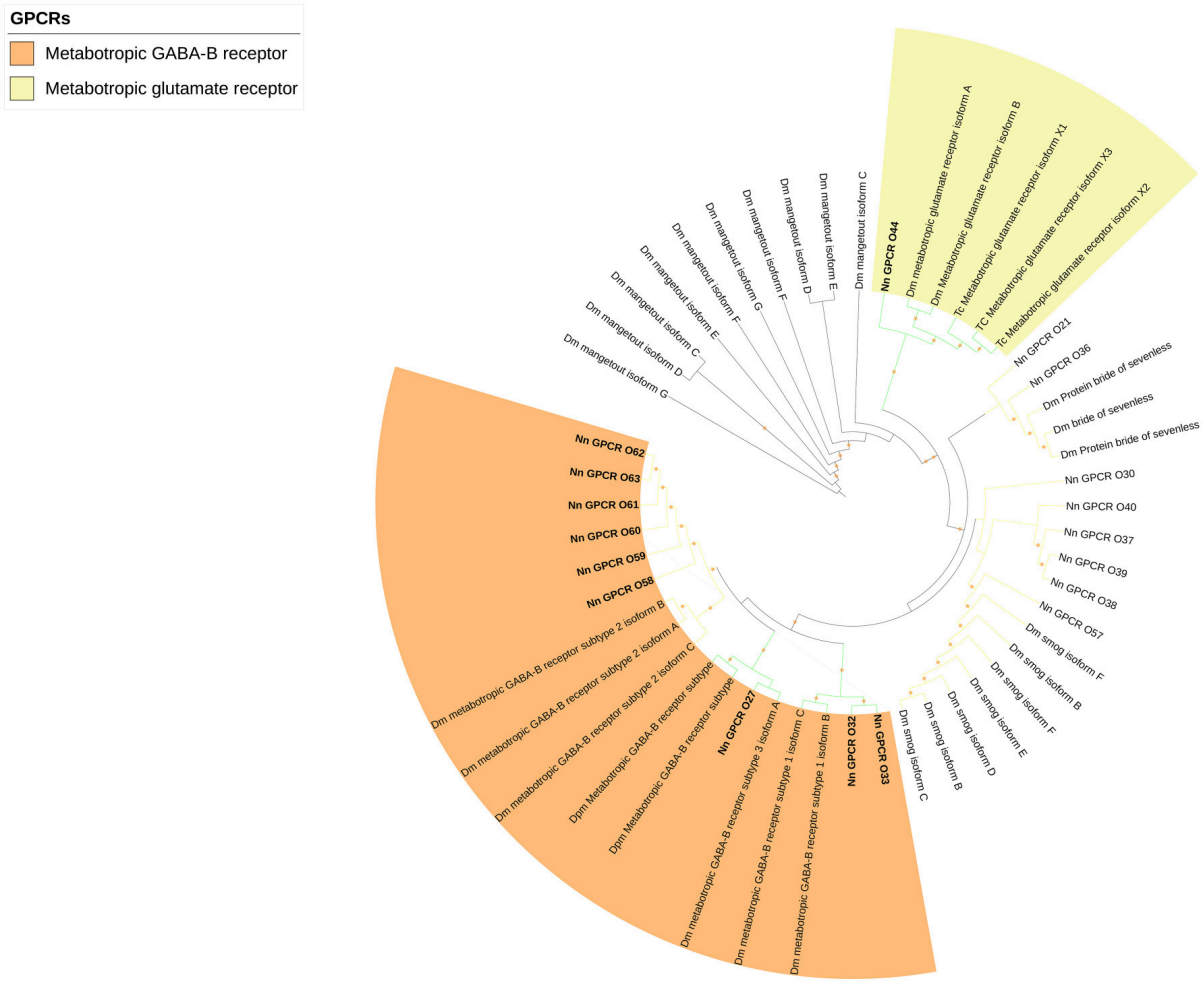
Phylogenetic tree of other GPCRs of *N. norvegicus* and other invertebrate species. *N. norvegicus* GPCR(s) clustering with high confidence are highlighted in bold. Green line: Clade annotated with high confidence. Yellow line: Clade annotated with low confidence. Red line: Unannotated clade. Orange stars represent clades with bootstrap value larger than 70. Dm, *Drosophila melanogaster*; Tc, *Tribolium castaneum*.

The rest of the uncharacterized GPCR families were grouped together to make the third group (Figure 5), which consisted of metabotropic glutamate receptor (7tm_3) and multiple unknown GPCRs (with Pfam domain 7tm_7, 7tm_Srx, 7tm_Srw). These GPCRs are very distinct and difficult to properly annotate since there are no previously deorphanized homolog GPCRs from other species.

### Details regarding neuropeptidergic systems involved in reproductive functions

**Figure 6:**
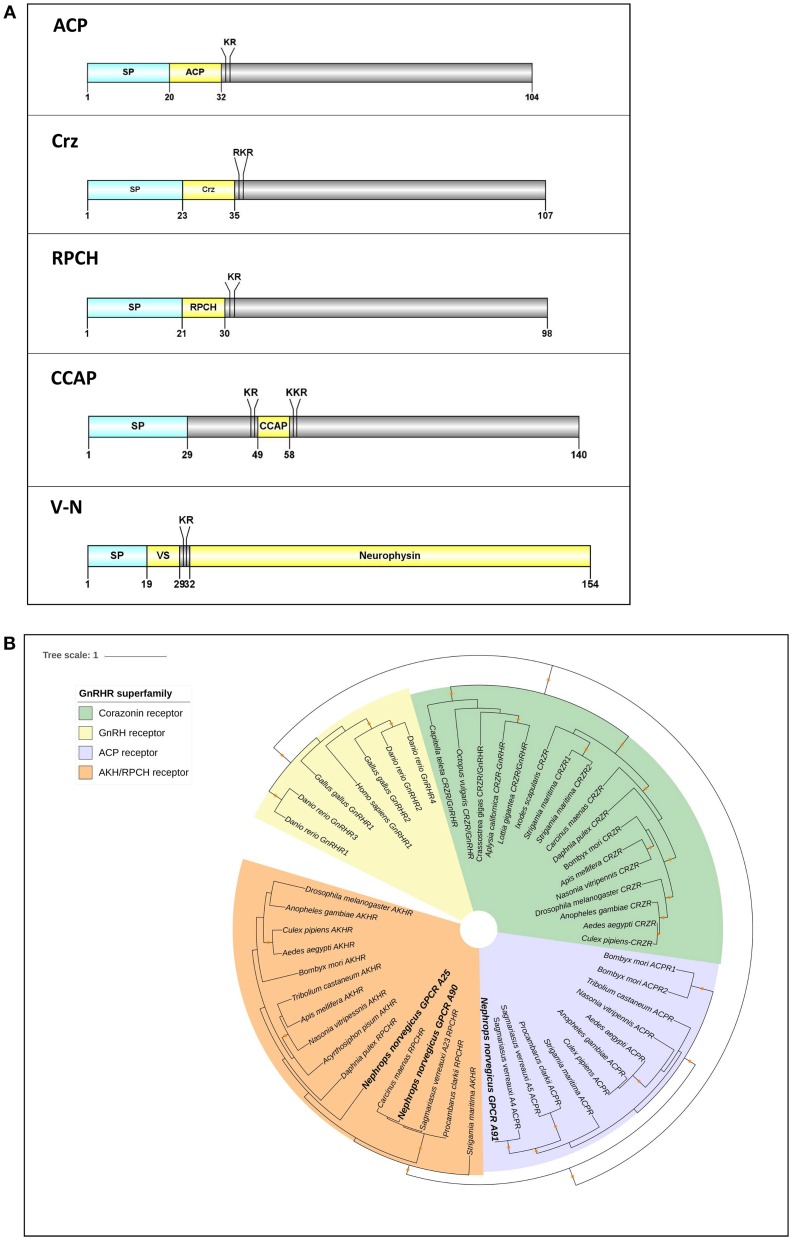
Analyses of neuropeptide and GPCRs in the GnRH superfamily (ACP, Crz, RPCH), V-N, and CCAP). **(A)** Illustration of neuropeptides ACP, Crz, RPCH, V-N, and CCAP. Schematic diagrams show organization of neuropeptide precursors, including signal peptide (SP), the mature peptide (yellow) and putative cleavage sites. ACP, Adipokinetic hormone/Corazonin-related peptide, Crz, Corazonin, RPCH, Red pigment concentrating hormone, CCAP, Crustacean cardioactive peptide, V-N, Vasopressin (VS)– Neurophysin. **(B)** Molecular phylogenetic analysis of GnRH-superfamily receptor by Neighbor-joining method based on the JTT matrix-based model. 1000 bootstrap replicates were used to produce the phylogenetic tree using amino acids sequence of GPCRs. The tree is drawn with branch lengths measured in the number of substitutions per site. Orange stars represent clades with bootstrap value larger than 70. *N. norvegicus* putative GPCRs are highlighted in bold font. ACP, Adipokinetic hormone/Corazonin-related peptide; GnRH, Gonadotropin releasing hormone; RPCH, Red pigment concentrating hormone.

We identified 3 members of the GnRH superfamily (Figure 6A). All have preprohormone that contain a signal peptide and cleavage sites for the release of a mature neuropeptide consisting of: ACP, qQITFSRSWVPQamide; Corazonin (Crz), pQTFQYSRGWTNamide; RPCH, pQLNFSPGWamide. In addition, there are 2 full-length neuropeptides whose receptors share a common ancestor with the GnRHR superfamily: CCAP and V-N (Figure 6A). Our phylogenetic analysis (see Figure 3) had revealed 3 putative GPCRs annotated as ACP/Crz/GnRH/RPCH GPCRs, which were further analyzed at a greater phylogenetic resolution (Figure 6B). This analysis combined with a previous GPCR analysis (Figure 3), allowed us to confidently predict 1 putative ACP GPCR and 2 putative RPCH GPCRs, 1 CCAP, and 2 V-N GPCRs (summarized in Supplementary Material S8). In addition, we also identified a fragmented Crz/GnRH GPCR that was not detected using our designated threshold. The Crz GPCR identified is fragmented into two partial fragments, one consists of 3 transmembrane helices and another with 4, thus could not be detected with our current threshold (Supplementary Material S8).

**Figure 7:**
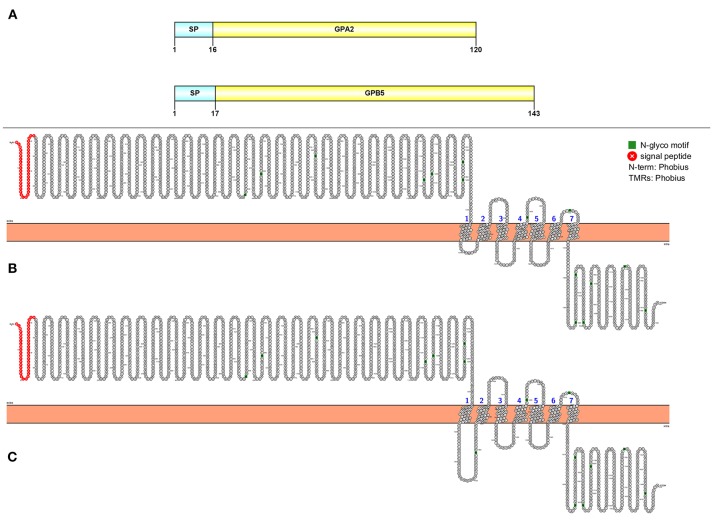
Molecular characterization of GPA2/GPB5 **(A)** and 2 GPA2/GPB5 putative GPCRs **(B, C)**. Schematic diagrams (above) show organization of neuropeptide precursors, including signal peptide (SP), the mature peptide (yellow). Schematic diagrams of receptor show illustration of putative GPCR(s), N-glycosylation motif amino acids (green), signal peptide (red), transmembrane prediction algorithm was conducted by Phobius (default setting).

Two full-length transcripts that encode GPA2 and GBP5 were detected in our study (Figure 7A). The GPA2 precursor is 120 aa, has a signal peptide followed immediately by the mature peptide. The GPB5 precursor is 143 aa in length, consisting of a 17 aa signal peptide and a 126 aa mature peptide. From our *de novo* assembly, we detected two putative GPA2/GPB5 GPCRs with very high confidence (Figures 7B,C).

**Figure 8:**
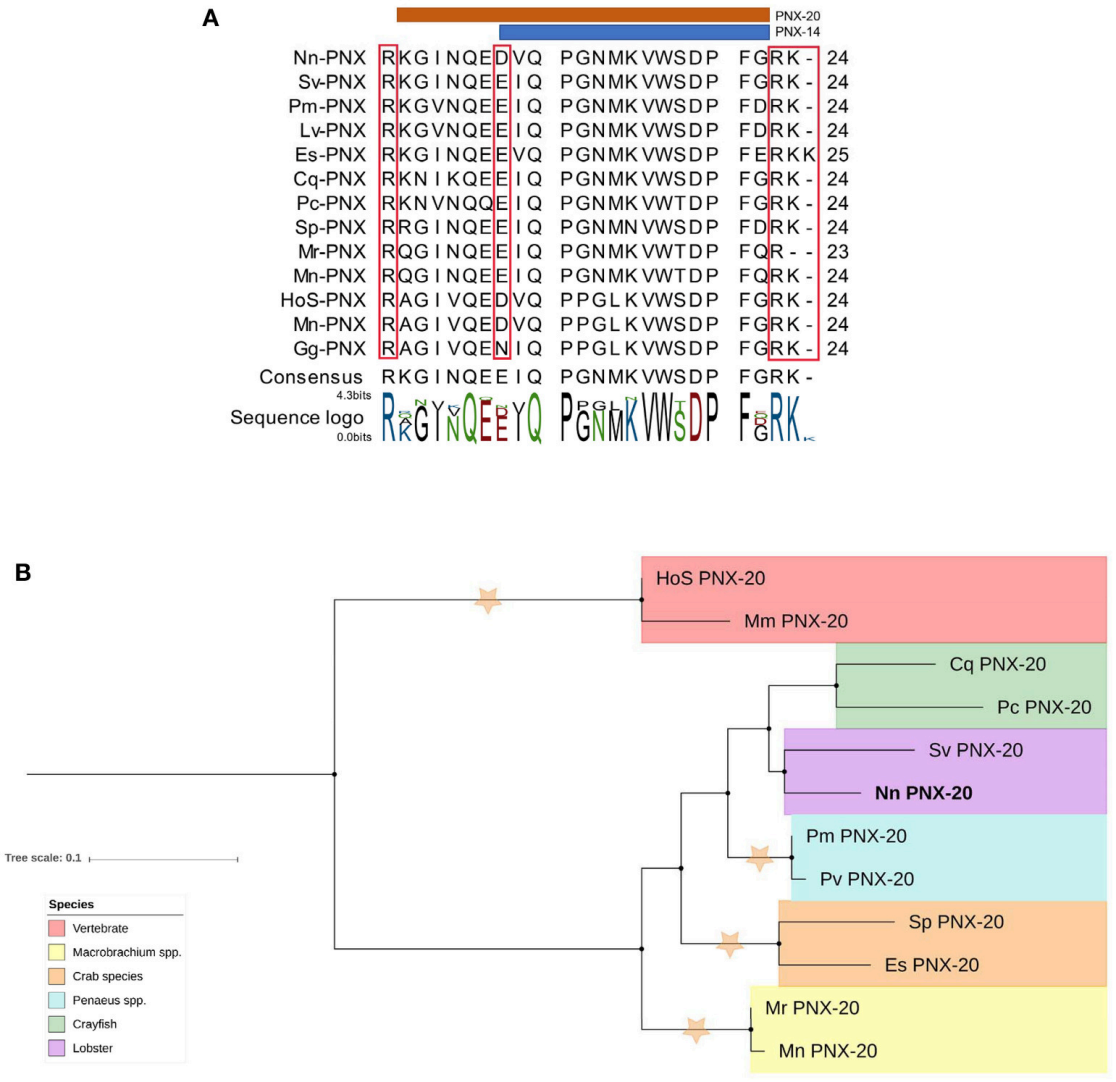
Analysis of PNX mature peptide between vertebrate and multiple decapod crustacean species. **(A)** Multiple alignment of PNX mature peptides,including the putative cleavage sites (red boxes). Orange and blue line illustrate putative mature peptide of PNX-14 and PNX-20. Cq, *Cherax quadricarinatus*; Pc, *Procambarus clarkii*; Sv, *Sagmarius verreauxi*; Nn, *Nephrops norvegicus*; Pm *Penaeus monodon*; Pv, *Penaeus vannamei*; Sp, *Scylla paramamosain*; Es, *Eriochier sinensis*; Mr, *Macrobrachium rosenbergii*; Mn, *Macrobrachium nipponese*; Hos, *Homo sapiens*; Mn, *Mus musculus*; Gg, *Gallus gallus*.**(B)** Molecular phylogenetic analysis of PNX by Neighbor-joining method based on the JTT matrix-based model. 1000 bootstrap replicates were used to produce the phylogenetic tree using amino acid sequences of the mature PNX peptides. The tree is drawn with branch lengths measured in the number of substitutions per site. Orange stars represent clades with bootstrap value larger than 70. *N. norvegicus* PNX are highlighted in bold font. Cq, *Cherax quadricarinatus*; Pc, *Procambarus clarkii*; Sv, *Sagmarius verreauxi*; Nn, *Nephrops norvegicus*; Pm, *Penaeus monodon*; Pv, *Penaeus vannamei*; Sp, *Scylla paramamosain*; Es, *Eriochier sinensis*; Mr, *Macrobrachium rosenbergii*; Mn, *Macrobrachium nipponese*; Hos, *Homo sapiens*; Mn, *Mus musculus*.

A putative phoenixin (PNX) neuropeptide was predicted from our *de novo* assembly. Similar to previously detected PNX in vertebrate species, *N. norvegicus* PNX does not contain a signal peptide. The full-length sequence is 70 aa with two cleavage sites that release a 20 aa mature peptide; one mature peptide of 14 aa is predicted (Figure 8A). Tissue screening by RT-PCR showed that PNX appears in all tissues tested in both mature and immature females (see Figure 3). In addition, we could retrieve PNX precursor transcripts from several other crustacean species through transcriptome mining of public and in-house data (Supplementary Material S9). A phylogenetic tree was constructed to investigate the conservation of PNX throughout both invertebrate and vertebrate species lineages (Figure 8B). The phylogenetic tree illustrates that the crustacean PNX are clustered to form their own clade with high confidence, although they all show quite substantial similarity with the vertebrate PNX, especially at the mature peptide region (See multiple alignment illustration in Figure 8A).

## Discussion

This is the first study to characterize the neuropeptidome and putative GPCRs of *N. norvegicus*. From our *de novo* transcriptome assembly, 57 different neuropeptide precursors were identified; the majority of these have also been found in other crustacean species. Importantly, we show for the first time in an invertebrate that the PNX neuropeptide is present. PNX was recently characterized in a few vertebrate species and found to be abundantly produced in the hypothalamus where it has important roles in regulating the ovarian cycle (61, 62).

Three neuropeptide transcripts previously identified in decapod crustacean species were not detected in *N. norvegicus*, including allatotropin (47), crustacean female sex hormone (CFSH) (44–46, 63), and the HIGSLYamide precursor (40, 45). This is interesting to note since our *N. norvegicus de novo* assembly used similar tissue types, as well as reproductive stages, to a previous study that identified these neuropeptides in another Nepropidae species (*C. quadricarinatus*) (45). Perhaps differences in their habitats (freshwater versus open sea) and life cycle (direct versus larval phases) could explain the absence of these neuropeptides in *N. norvegicus*, although deeper sequencing could be required to identify them.

Our study predicted 223 different GPCRs, which is a number consistent with other decapod crustacean-based studies (48, 49, 64). The majority of *N. norvegicus* GPCRs could be placed confidently within the Rhodopsin superfamily (Pfam domain 7tm_1) and Secretin superfamily (Pfam domain 7tm_2), yet 63 GPCRs (Pfam domain not 7tm_1 or 7tm_2) were placed into a third group. Information relating to neuropeptide annotation of these GPCRs is currently vague and hard to confidently annotate without close homologs of known function in closely related species. In addition, we could not detect several known neuropeptide GPCRs that were previously found in other arthropods (for instance Crz, sulfakinin, allatostatin-B, and pyrokinin receptors), perhaps due to the stringency of our detection threshold (as exemplified by our identification of the truncated Crz receptor (Figure 6A).

Based on RNA-seq expression of neuropeptide, the majority are abundantly expressed in the CNS. Interestingly, more neuropeptides are expressed in the ovary at the immature stage compared to the mature stage, whereas the opposite is observed in the hepatopancreas known as the major site of vitellogenin production in crustaceans outside of ovary [reviewed by Subramoniam (65)]. We hypothesize that neuropeptides that are expressed in the immature ovary may have roles in preparation for maturation, neuropeptides then shift in balance from ovary during ovarian maturation to the hepatopancreas in mature individuals, where the hepatopancreas could potentially serve as a reservoir for neuropeptides, ready for the next maturation cycle. The current study listed all the identified neuropeptides and GPCRs with a specific focus given to those that are plausibly related to sexual maturation and reproduction. This shortlisted catalog of candidate neuropeptides and their putative receptors can now serve as a starting point for further investigation. For example, effect of these neuropeptides and GPCRs on ovarian maturation throughout the reproduction cycle of *N. norvegicus* (and other related crustacean species) could be explored using *in vitro* and *in vivo* bioassays.

The GnRH, ACP, Crz, and RPCH are clustered together in a GnRH-like superfamily of neuropeptides (60). In bilaterians, the receptors for these neuropeptides are closely related to the receptors for CCAP and V-N (also known as oxytocin-vasopressin in vertebrates) (66). In addition, a few studies have been attempted to link the homologs of “GNRH-like” across invertebrate and vertebrate species at both receptor(s) and ligand(s) levels (67–69). Our phylogenetic analysis confirmed this relatedness at the receptor level, where one clade was formed that included all the above receptors (see Figure 4).

GnRH is known to be a key stimulant in vertebrate reproduction; however, in arthropods the function of GnRH-like peptides is currently unclear. Several studies have identified GnRH-like peptides in decapod species, including *Penaeus monodon* (23), *Penaeus vannamei* (24), *M. rosenbergii* (22, 26), and *Procambarus clarkii* (25). However, injection of GnRH-like peptides does not affect ovarian maturation (25, 26). With the rapid increase in decapod sequence databases available for analysis, several recent studies have highlighted the absence of GnRH (49). This absence could be explained by: (a) GnRH/GnRH-like is stage-specific, and transcriptomic analysis has not been conducted on the appropriate stage, (b) GnRH/GnRH-like is not present in all crustacean species, and those GnRH identified in crustaceans have functions unrelated to reproduction, or (c) AKH and ACP represents a class of GnRH-like that have arisen by gene duplication in the arthropod lineage, as elucidated recently by Zandawala et al. (69). One of the closest related neuropeptides to GnRH, in terms of primary amino acid sequence, is Crz, a neuropeptide which was originally assigned as a cardioactive peptide (70), and then has been proven to hold a wide range of functions including within the context of stress (71–73), pigmentation (74), ecdysis (75), and circadian rhythm (76). As reviewed recently, the role of Crz in crustaceans reproduction is still uncertain (21). Crz was shown to elicit an inhibitory effect on the androgenic gland and therefore, reduced masculinity in *M. rosenbergii* (77, 78). In terms of receptor, Crz receptor was recently deorphanized in the Green Shore Crab, *Carcinus maenas* (79). Research to investigate the precises role of Crz in crustacean reproductive biology is therefore, warranted.

In addition, the RPCH could be identified from our *de novo* assembly and showed expression in the CNS but not in ovary or hepatopancreas tissues of mature or immature females. Many recent studies all point toward a role of RPCH in reproduction or sexual maturation in various taxonomic groups. For example, RPCH was shown to accelerate gonadal maturation in *P. clarkii* as determined by both *in vitro* and *in vivo* experiments (80, 81). Recently, in the mud crab *Scylla paramamosain* it was shown that RPCH could stimulate ovarian maturation, possibly through a stimulatory effect on the nervous tissues (32). Also, another study has shown that serotonin can induce gonad maturation in *P. monodon* (82), which later on elucidated that the phenomenon can be achieve through upregulation of RPCH gene expression (83). Most recently, an RPCH receptor has been deorphanized in *C. maenas* that is expressed in the ovary (79).

In *N. norvegicus*, ACP can be found in the CNS tissues as well as the ovary (immature and mature female) and hepatopancreas (mature female). The role of ACP is not yet clear in crustacean species. In female *M. rosenbergii, in vivo* bioassay indicate that ACP has no effect on ovarian maturation except at a high concentration (500 ng/g), which reduces the rate of germ cell proliferation (26).

While members of the GnRH family have been implicated in the regulation of reproduction in vertebrate and some invertebrate, including crustaceans, the neuropeptides that share receptor evolution with GnRH (CCAP and V-N) have not been assigned a role in reproduction yet. Although being evolutionary linked at the receptor sequence level, the neuropeptides are quite distinct from other GnRH superfamily members (i.e., ACP, RPCH, Crz) in sequence. The CCAP has been found in multiple crustacean species including *C. quadricarinatus* (45), *S. paramamosain* (40), *M. rosenbergii* (43) and *P. clarkii* (44, 49). Our multi-tissue RT-PCR, which detected expression of CCAP in the ovary of immature female *N. norvegicus*, is consistent with its observed expression in *C. quadricarinatus* (45) and *M. rosenbergii* (43), indicative of the fact that CCAP can be expressed in the ovary of multiple crustacean species. Although the presence of CCAP in ovaries of various crustacean species, the only CCAP function demonstrated in crustaceans is cardio-activity (84–86), and it is also a modulator of oviduct contraction in the insect *Locusta migratoria* (87). Similarly, the precise role of V-N has not been clearly established in any crustacean species. Prediction of both ligands as well as the binding receptors of both CCAP and V-N might be valuable to characterize its function, not only in *N. norvegicus*, but also in closely related crustacean species as *C. quadricarinatus* and *P. clarkii*.

*In silico* expression analysis (and also confirmed by RT-PCR) demonstrated that only GPB5 is expressed in the ovary of immature *N. norvegicus*, similar to that found in *C. maneas* (49), and *P. clarkii* (44). GPA2 and GPB5 are related to the heterodimeric glycoprotein thyrostimulin, from which the vertebrate Luteinizing hormone (LH)/Follicle-stimulating hormone (FSH)/Thyroid Stimulating Hormone (TSH) evolved (88–90). In general, GPA2- and GPB5-like subunits have been identified or predicted in a wide array of animal phyla, from nematodes, arthropods, annelids and molluscs, through to echinoderms and chordates. Their precise role is still debated [reviewed by Rocco and Paluzzi (91)]. GPA2/GPB5 has been proposed to be a major factor in the search for the gonad stimulating factor in crustacean species (21). The GPA2/GPB5 glycoprotein binds to leucine-rich repeat-containing (LGR) GPCRs of *D. melanogaster* (92). The LGR1 is also deorphanized and GPA2/GPB5 was linked to ionic regulation and osmoregulation in the midgut of mosquito *Aedes aegypti* (93). Receptors for both proteins have a very long N-terminus region, which is also a feature of GPA2/GPB5 receptors in *Aedes aegypti, Daphnia pulex*, and *Chilo suppressalis* (data not shown). The long N-terminus includes a characteristic number of tandemly placed leucin-rich and lipoprotein domains, which are instrumental in characterizing and annotating these receptors (94).

Also, we highlighted some other neuropeptides of interest that perhaps might contribute toward reproduction of the species. Based on RT-PCR validation, pyrokinin, tachykinin as well as kinin are expressed in the ovary of the immature female *N. norvegicus*, but not mature females. This perhaps can be explained by the fact that females must accumulate nutrients in order to prepare for the ripening of the ovary, perhaps once activated, there is no longer a need for neuroendocrine factors to facilitate the process. PDH is a neuropeptide that has been previously suggested to be involved in regulation of pigment in the eyestalk of crustaceans (15). Moreover, recent studies report the involvement of PDH in regulation of ovarian cycle (35, 40). We could detect three different isoforms of PDH preprohormone from *N. norvegicus de novo* assembly and also multiple putative PDH GPCRs that cluster with insect Pigment dispersing factor (PDF) homologs (see Figure 4). Another important neuropeptide family that might be involved in crustacean reproduction is the CHH-superfamily. Using our designated threshold, we could identify several members of CHH, as well as a newly described CHH-like neuropeptide. Similar to the *in silico* FPKM calculation, RT-PCR amplification of CHH-like indicates expression in multiple tissues in *N. norvegicus* (both in mature and immature individuals), suggesting an important regulatory role of this newly characterized CHH-like. Another candidate neuropeptide that might have a potential role in reproduction and sexual maturation is neuroparsin. From our *de novo* assembly, we were able to deduce 4 different neuroparsins (namely NP-1, 2, 3, and 4). FPKM values show that most neuroparsins are expressed in the ovary at the immature stage, while it cannot be detected in ovary of mature individuals. Neuroparsins have roles in reproduction and development in crustaceans (33, 95, 96). Recently, a shrimp neuroparsin was confirmed to be involved in regulating the initial stage of vitellogenesis (33). There is not yet a confirmed GPCR for neuroparsin in crustaceans; a study in mosquito suggest that GPCR is a Venus Kinase receptor (97). Further research is therefore warranted to better understand the role of these precursors and their putative GPCRs in reproduction in *N. norvegicus* and crustaceans in general.

Lastly, phoenixin (PNX) is a neuropeptide that was initially discovered in vertebrates in 2013 (61). In *N. norvegicus*, we found *PNX* in the CNS tissues as well as other tissues, consistent with the wide distribution of *PNX* in the brain and peripheral regions in vertebrates. Similar to vertebrates, the decapod crustacean PNX precursor predicted in the current study can yield amidated neuropeptides with different isoforms (in vertebrates, at least two active forms can be found, namely PNX-14, PNX-20–See Figure 8B) (61, 62). PNX has been identified as a reproductive peptide in vertebrates (61, 62, 98). Pre-treatment of female rat primary anterior pituitary cells with 1000 nM Phoenixin-20 amidated peptide significantly, although temporarily, increases GnRH-stimulated LH release (61). Also in the same study, siRNA silencing of PNX resulted in the delayed appearance of oestrus and decline in GnRH receptor expression (61). Data also shows that during the rat estrogenous cycle, when estrogens are at their lowest level, PNX mRNA level is significantly upregulated (99). PNX was found in all the studied tissues of *N. norvegicus* showing a high expression in the ovaries of mature and immature females and the hepatopancreas of mature females, suggesting potential implication of PNX in oocyte maturation. Recently, PNX was found to activate GnRH and kisspeptin neurons through a novel GPCR named GPR173 (98). However, we could not find any related GPCR in this study with the designated threshold, suggesting that while the neuropeptide retains its structure, the putative PNX GPCR has diverged considerably.

## Conclusions

This study characterized putative neuropeptide-encoding transcripts as well as GPCRs in the commercially important crustacean species *N. norvegicus*. Through bioinformatics analyses, we revealed several neuropeptide-encoding transcripts that are known to be involved in reproduction and development in crustaceans. This study broadens the molecular toolbox needed for further research of crustacean reproduction, including the first discovery of Phoenixin in an invertebrate. We anticipate that the generated dataset could lead to extended understanding of reproduction in *N. norvegicus*, promoting future hatchery technology in the species to enhance fishery through restocking programmes.

## Author contributions

TN, GR, AE, SC, and TV conceived and designed the experiments. TN and GR performed the experiments. TN, GR, and TV analyzed the data. TN wrote the paper. All authors proofread and accepted the final manuscript.

### Conflict of interest statement

The authors declare that the research was conducted in the absence of any commercial or financial relationships that could be construed as a potential conflict of interest.

## Supplementary material

Supplementary Material S1 List of primers used.

Supplementary Material S2 Full RT-PCR gel image(s).

Supplementary Material S3 GenBank accession number and all used sequences in this study.

Supplementary Material S4 Comparative list of neuropeptides detected in previous studies.

Supplementary Material S5 Schematics of all detected neuropeptides.

Supplementary Material S6 Amino acid sequences of all detected neuropeptides.

Supplementary Material S7 List of all N. norvegicus GPCRs detected in this study.

Supplementary Material S8 Illustrations of ACPR, CrzR, RPCHR, CCAPR and V-NR.

Supplementary Material S9 Phoenixin sequences used in this study.
