# Supplementary material for: Insights Into Sexual Maturation and Reproduction in the Norway Lobster (Nephrops norvegicus) via in silico Prediction and Characterization of Neuropeptides and G Protein-coupled Receptors
Source: Front Endocrinol (Lausanne). 2018 Jul 27;9:430. doi: 10.3389/fendo.2018.00430 (PMC6073857; doi:10.3389/fendo.2018.00430)
Supplement: Supplementary Material S1 — List of primers used. [file Data_Sheet_1.docx]

**Table S1**: Primers used in the current study, CCAP: Crustacean cardioactive peptide, CHH: Crustacean hyperglycemic hormone, GPA2: Glycoprotein Alpha 2, GPB5: Glycoprotein Beta 5, PNX: Phoenixin, GADPH: Glyceraldehyde 3-phosphatedehydrogenase

| **Neuropeptide** | **Forward primer** | **Reverse primer** |
| --- | --- | --- |
| Nn_CCAP | TGACTTGCTGGAGGGTAAA | TTGTTGAGGTGTGGAGGTGA |
| Nn-CHH-like | GCAATCTGTTCTCCATCCAAA | AACGCTGCTCTCTTATTTAGGC |
| Nn_Kinin | GGATTTGTTTGTGTTGGATGG | GTCGTATTTGGCGTCCTGTT |
| Nn_Pyrokinin | CGGCAAGAGAGGTGATGGTT | TAGGGCTGAAGGCGAAAT |
| Nn_Tachykinin | CTGTCCCCTGGAAAACCAA | TTCTTGCCCCTCATACCC |
| Nn_GPA2 | TCGTATCAGCATCCCAGAGT | TCACCTTCACCTTCACATCC |
| Nn_GPB5 | GAGCCATCAACCCTCAGTCTA | GAGTCGCTTCGTGGTAAGAGT |
| Nn_SIFamide | CGAGTAGTGGTGGCGTTG | TTCTCTGGGACGGGGAAC |
| Nn_PNX | TCTCTTCGTTGGGGGTTTG | TCCCTTTCCTGGTTACTGCT |
| Nn_GADPH | AGTCCCCTCGCAACACCT | CCATCCTCCATCTTCACCTC |
