# Supplementary material for: Insights Into Sexual Maturation and Reproduction in the Norway Lobster (Nephrops norvegicus) via in silico Prediction and Characterization of Neuropeptides and G Protein-coupled Receptors
Source: Front Endocrinol (Lausanne). 2018 Jul 27;9:430. doi: 10.3389/fendo.2018.00430 (PMC6073857; doi:10.3389/fendo.2018.00430)

Suplementary S2: All gel photos

# GADPH

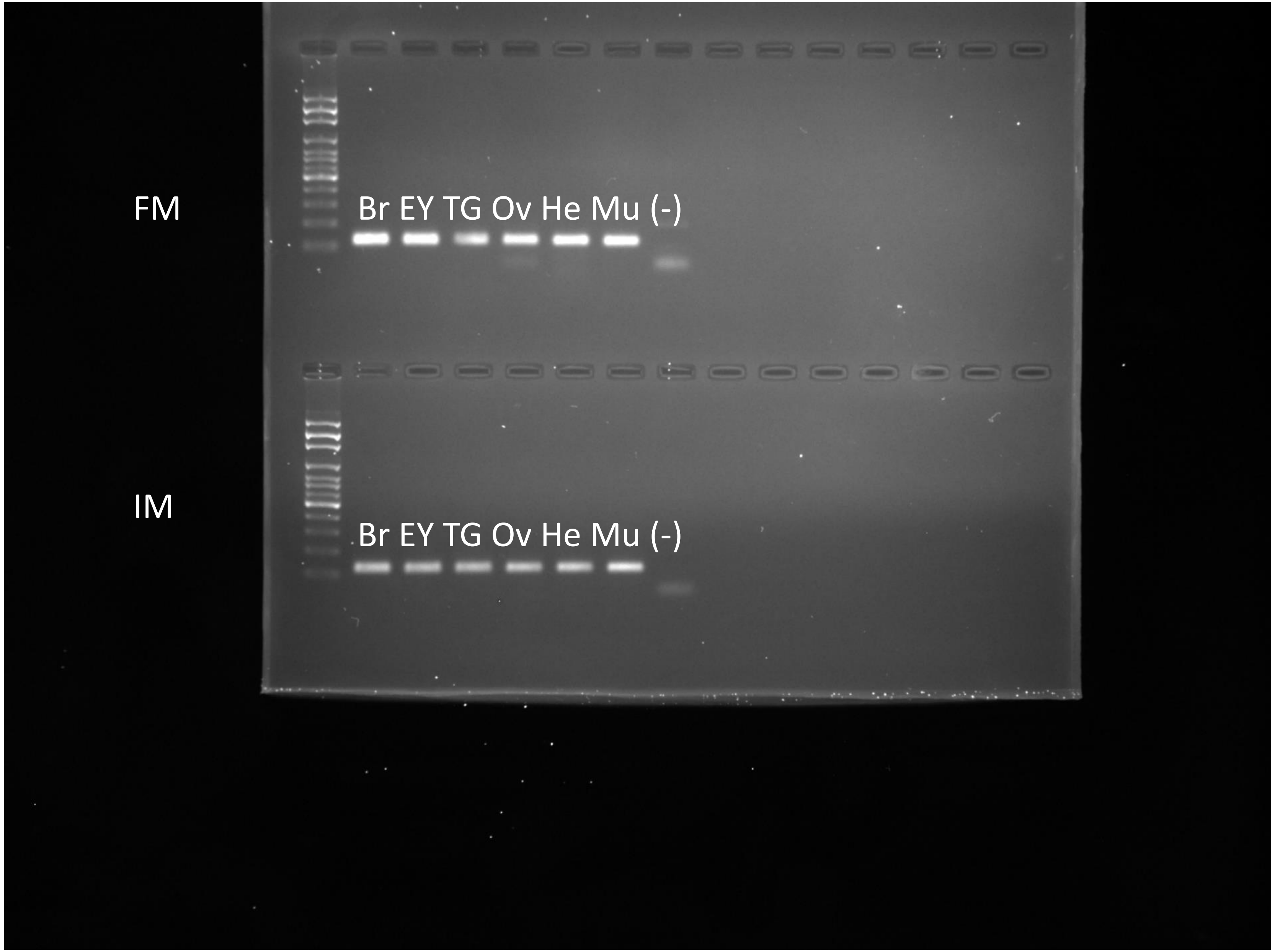

# CHH-like

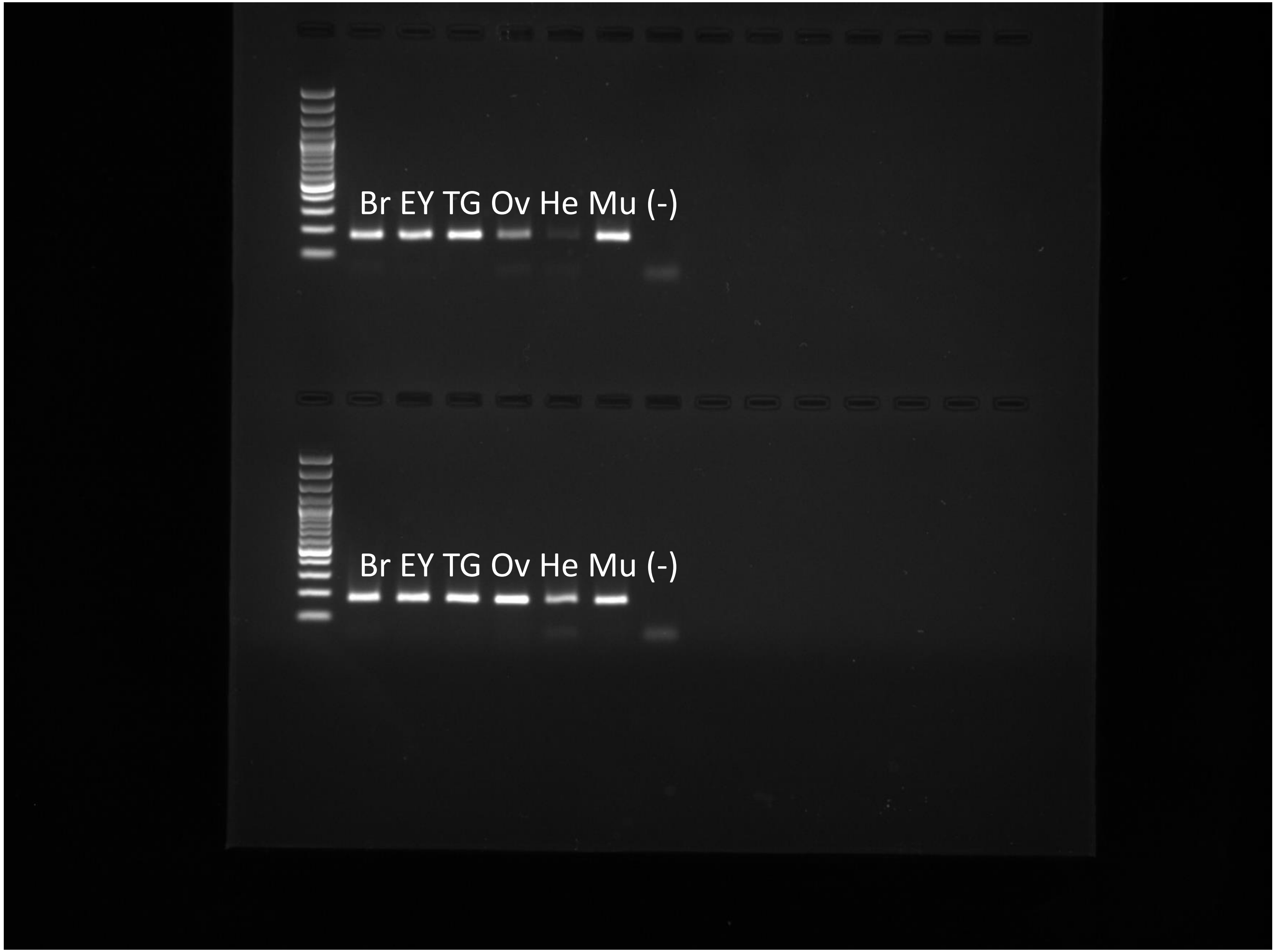

# GPA2

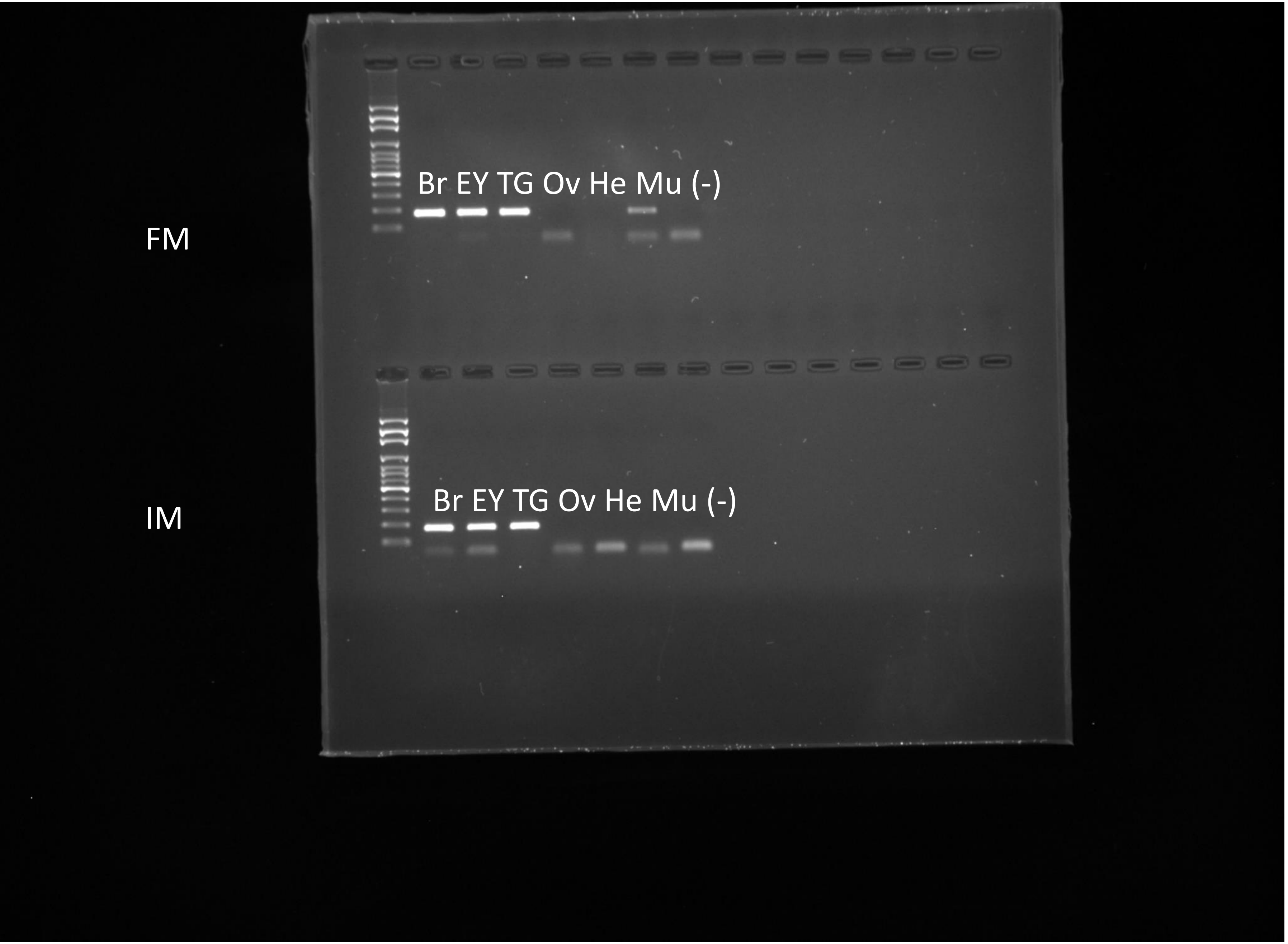

# GPB5

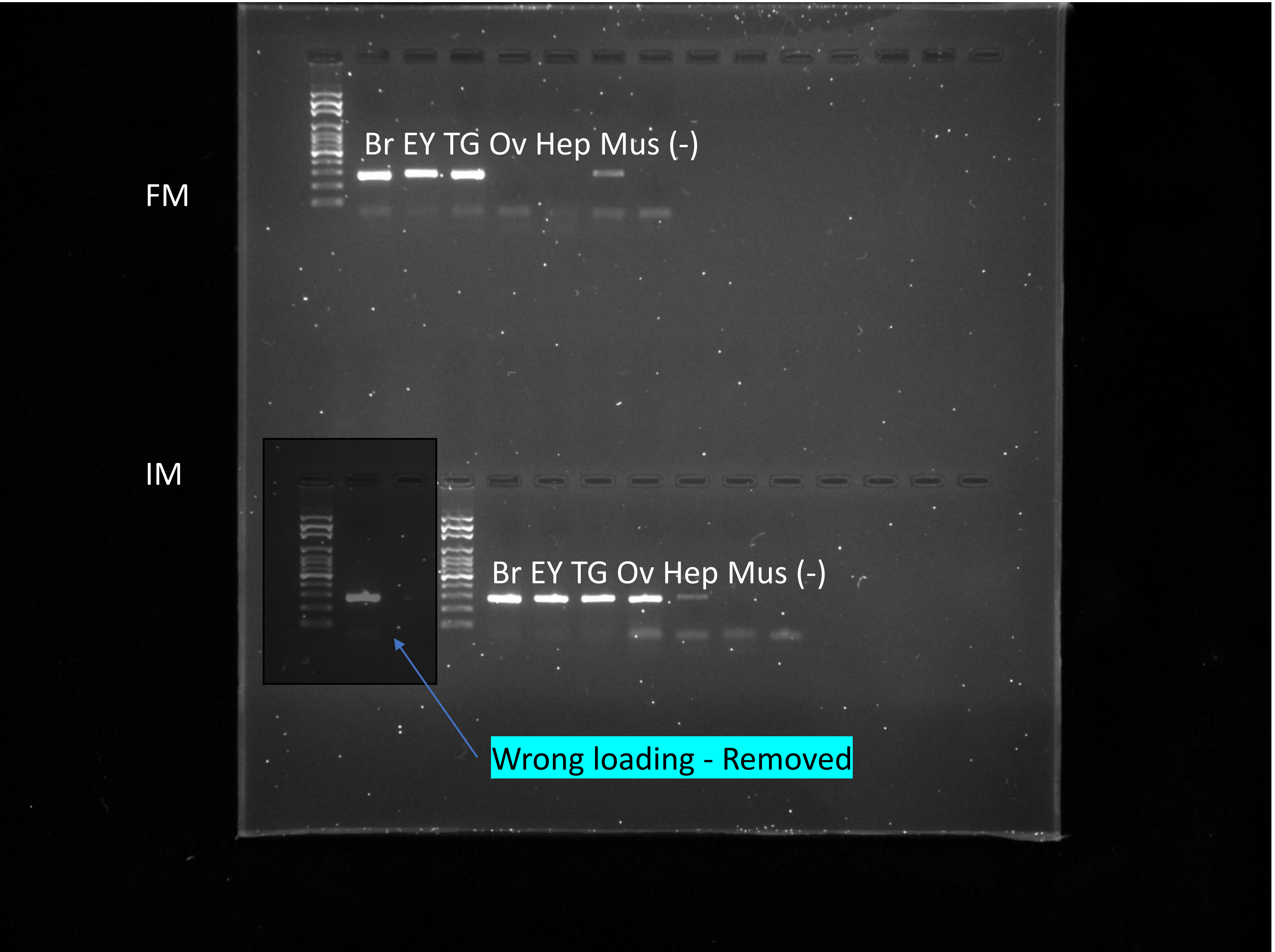
FM
IM
Br EY TG Ov Hep Mus (-)
Br EY TG Ov Hep Mus (-)
Wrong loading - Removed

# PNX

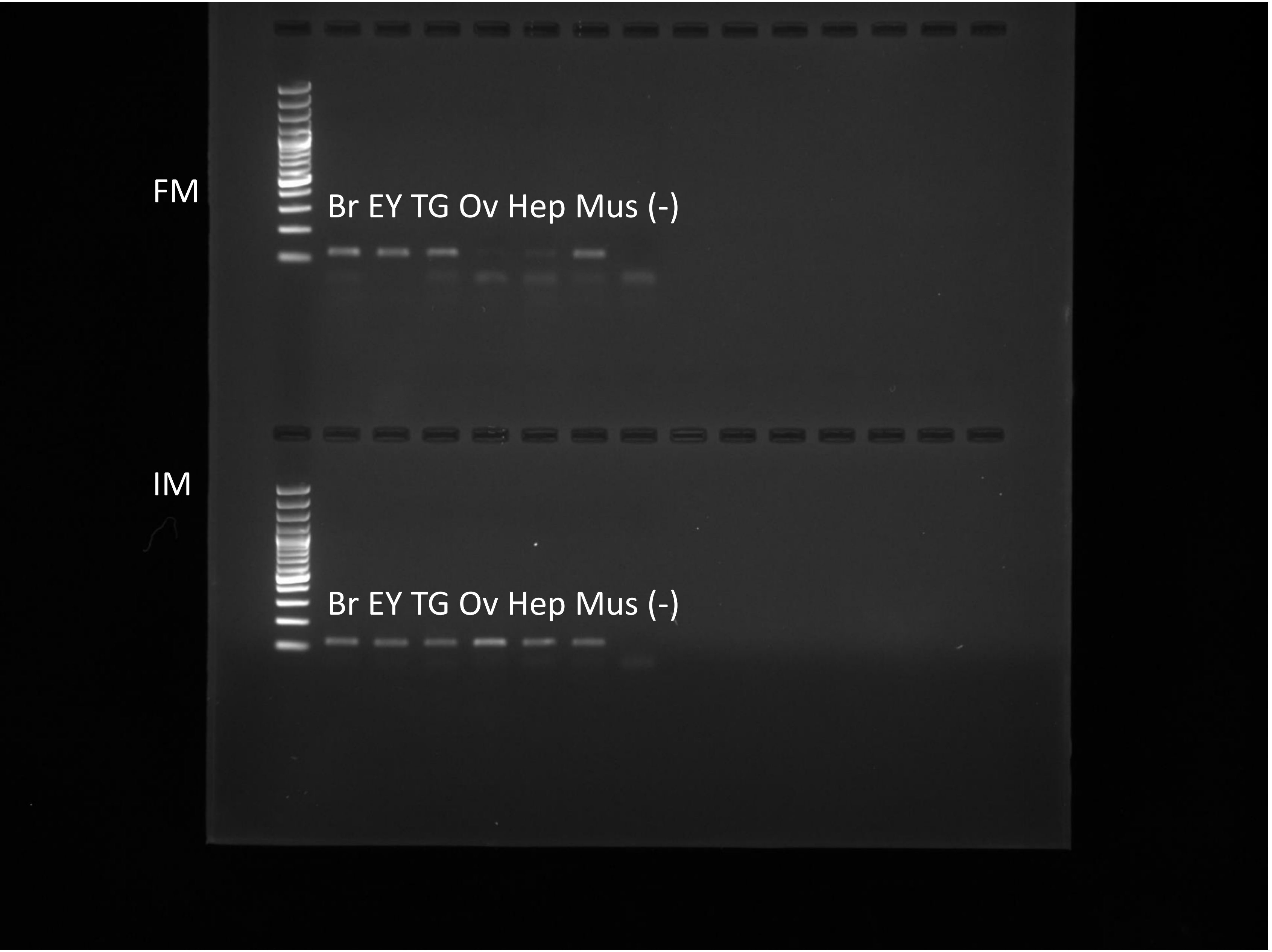

# SIFamide

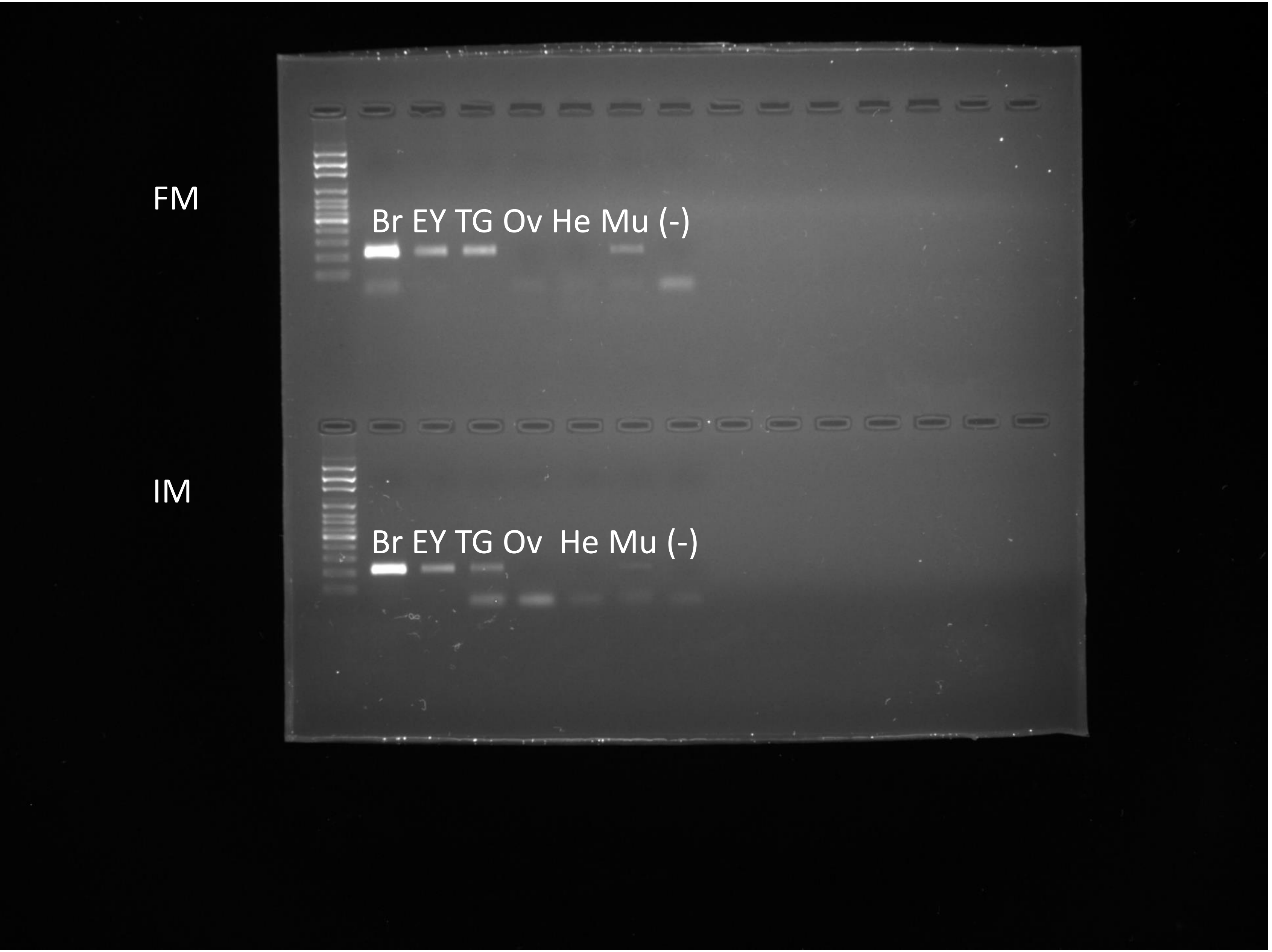

# Kinin

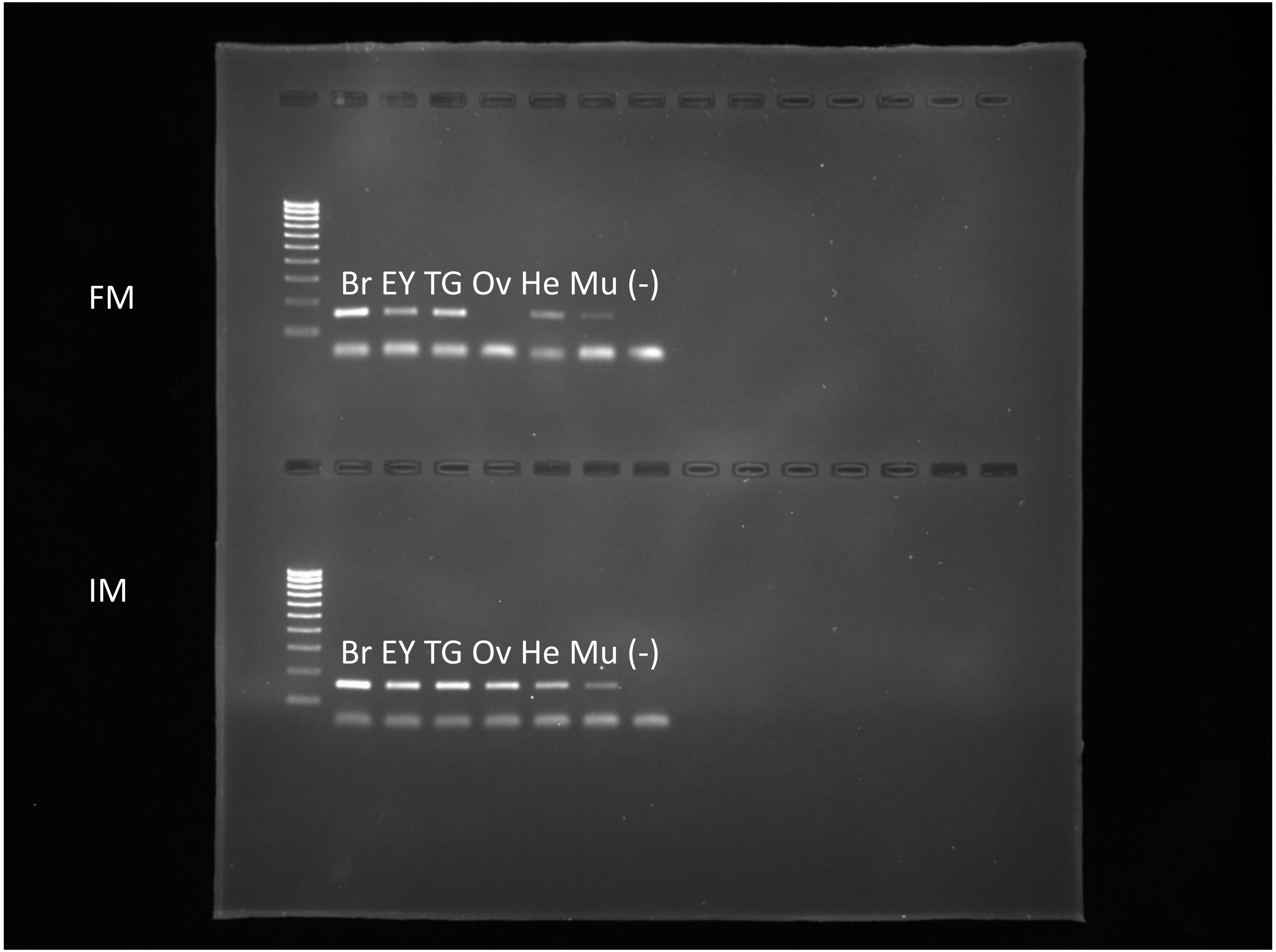

# Tachykinin

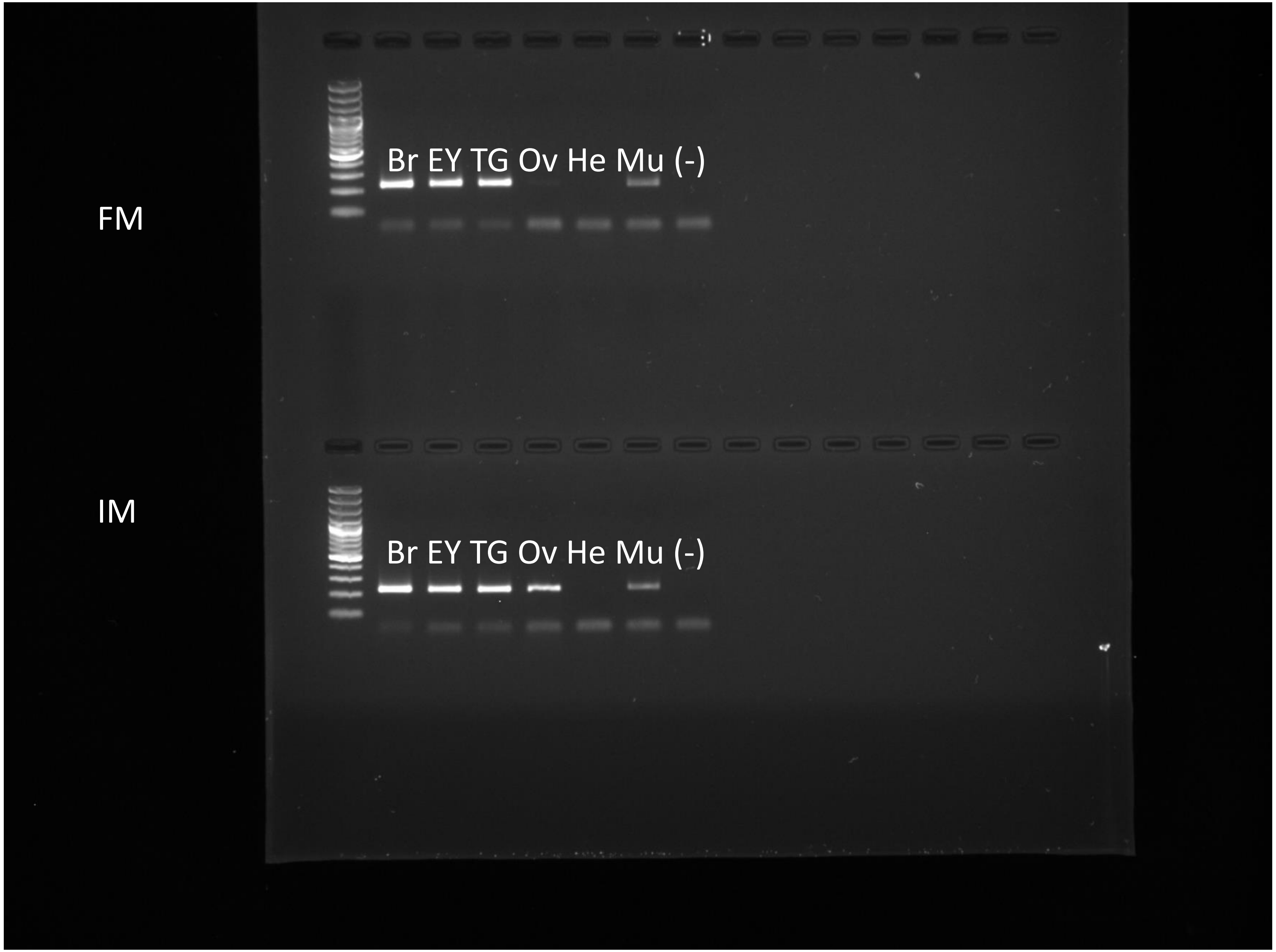

# Pyrokinin

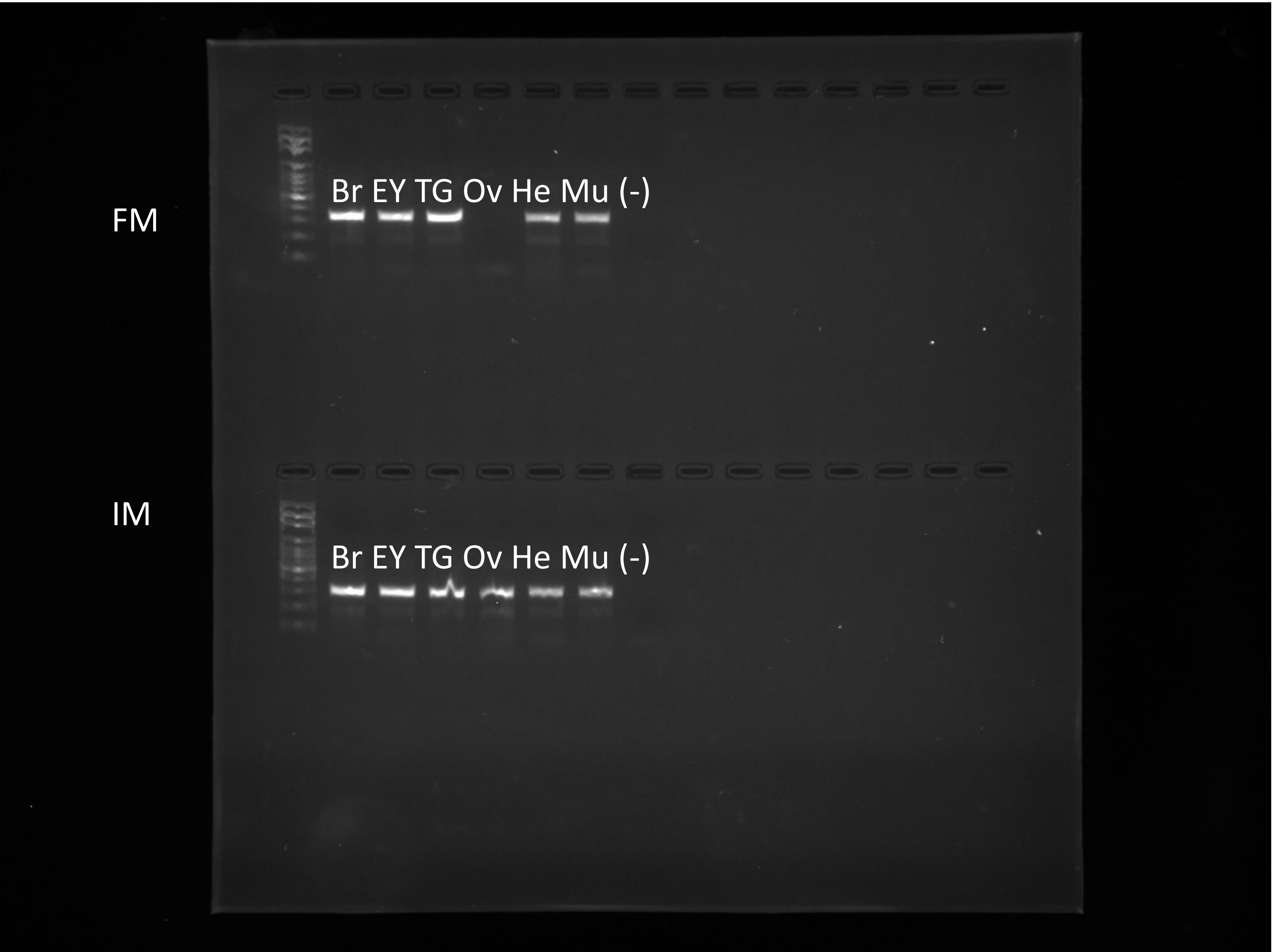

# CCAP

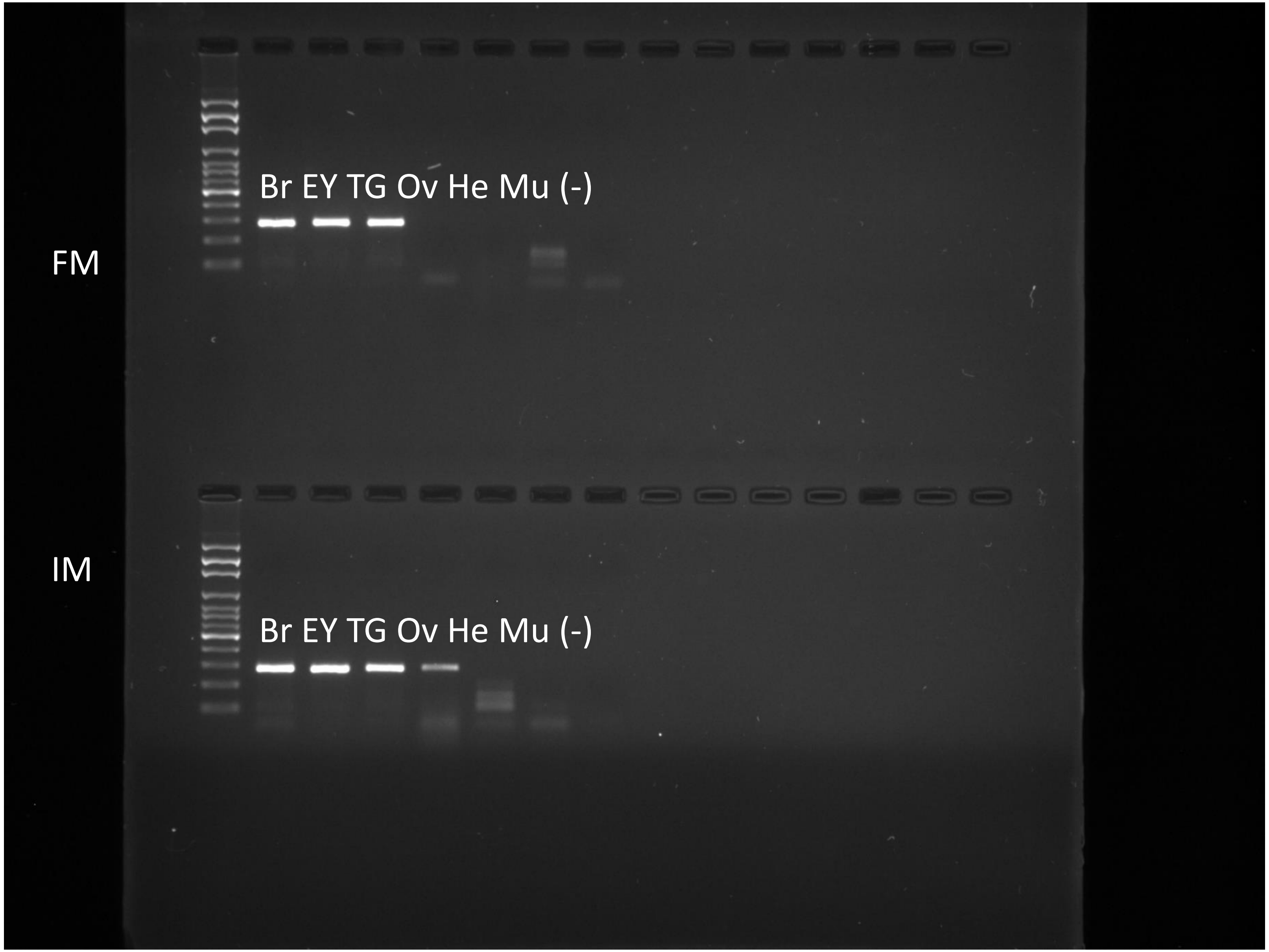

Supplement: Supplementary Material S2 — Full RT-PCR gel image(s). [file Data_Sheet_2.pdf]
