# Supplementary material for: Insights Into Sexual Maturation and Reproduction in the Norway Lobster (Nephrops norvegicus) via in silico Prediction and Characterization of Neuropeptides and G Protein-coupled Receptors
Source: Front Endocrinol (Lausanne). 2018 Jul 27;9:430. doi: 10.3389/fendo.2018.00430 (PMC6073857; doi:10.3389/fendo.2018.00430)
Supplement: Supplementary Material S4 — Comparative list of neuropeptides detected in previous studies. [file Data_Sheet_4.pdf]

	N. norgevicus (this study)	C. quadricarinatus (1)	P. clarkii (2)	H. americanus (3)	S. verreauxi (4)	M. rosenbergii (5)	L. vannamei (6)	S. paramamosain (7)	C. maenas (8)	E. sinensis (9)
ACP	A	A	A	A			A		A	A
Allatostatin-A	A	P	A	A	3	A	P	A	4	P
Allatostatin-B	A	A	P		A	A	A	A		A
Allatostatin-B1	P	A			P		A		2	A
Allatostatin-B2	P	A			P		A		2	A
Allatostatin-C	A	2	2	2	A		2		3	2
Allatostatin-cc		A	A				P			A
Allatotropin	A			A						
Bursicon-A	A	P	A	A	A	A	A	A	A	A
Bursicon-B	A		P	P		A	A	A	2	A
Calcitonin	A		A				2			2
CCAP	A	A		A		A	A	A	A	A
CCHamide-1	A	A	A	A			P		P	A
CCHamide-2	A	A	A	A			A		P	A
CCRFamide			A				P			P
CNMamide	A		A				A			P
Corazonin		A	A	A	P	A	A	A	A	A
CFSH			A		A		4			4
CFSH-like	4	A	2							
CHH	A	3	2	4	4	3	8	A	3	3
CHH-like	A	A	A				2			A
MIH		A	A		3		4		2	A
MIH-like	A	2	A			2		A		A
ITP		A								A
DH31	A	A		A	A				A	A
DH44	P	A	A	A			A		P	A
Eclosion hormone 1	A	A	P	A	A	A	A	A	A	A
Eclosion hormone 2	P		A	A	A		A		P	A
GSEFLamide	A	A	A	A			P			P
Elevenin	A		A				A			A
FMRFamide	A	A	A				A			A
GPA2	A	A	A				A			A
GPB5		A	A				2			A
HIGSLYRamide	A	P							A	
Kinin/Leucokinin	A	A	A	2			A		P	P
Myosuppressin	6	A	A	A		A	A	A	A	A
Neuroparsin	2	3	3	A	2	2	4	A	5	3
Neuropeptide F	A	2	2	A	A	3	3	A	2	3
Orcokinin	A	A	A	A	A	A	A	A	2	P
Periviscerokinin	3		A				A			P
PDH		3	3	1	2	5	A	A	5	3
Prohormone-1		A			P					
Prohormone-3					A					
Prohormone-4	A	A			A					
Proctolin	A	A	A	A			A		A	A
Pyrokinin	A	A	A	2			P		P	P
Relaxin	A		A	A			A			P
RPCH	A	A	A	A		A	A	A	A	A
Ryamide	A	A	A	A			A		A	A
sNPF	A	A	A	A			A		P (2)	A
SIFamide	A	A	A			A	A	A	A	A
Sulfakinin	A	A	A	A	A	A	P	A		A
Tachykinin	A	A	A	A	A	A	A	A	A	P
Trissin	A	A	A				A			A
Vasopressin-neurophysin	P	A	A			A	A	A		A
WXXXRamide		P	A							

Legends

A	Available
P	Partial sequences
	Undetected/not available
n	number of isoforms detected

References

1. Nguyen TV, Cummins SF, Elizur A, Ventura T. 2016. Transcriptomic characterization and curation of candidate neuropeptides regulating reproduction in the eyestalk ganglia of the Australian crayfish, *Cherax quadricarinatus*. Scientific reports 6: 38658.
2. Veenstra JA. 2015. The power of next-generation sequencing as illustrated by the neuropeptidome of the crayfish Procambarus clarkii. General and comparative endocrinology 224: 84-95.
3. Christie AE, Chi M, Lameyer TJ, Pascual MG, Shea DN, Stanhope ME, Schulz DJ, Dickinson PS. 2015. Neuropeptidergic signaling in the American lobster *Homarus americanus*: New insights from High-Throughput nucleotide sequencing. PloS one 10.
4. Ventura T, Cummins SF, Fitzgibbon Q, Battaglene S, Elizur A. 2014. Analysis of the central nervous system transcriptome of the eastern rock lobster *Sagmariasus verreauxi* reveals its putative neuropeptidome. PloS one 9.
5. Suwansa-ard S, Thongbuakaew T, Wang T, Zhao M, Elizur A, Hanna PJ, Sretarugsa P, Cummins SF, Sobhon P. 2015. In silico neuropeptidome of female *Macrobrachium rosenbergii* based on transcriptome and peptide mining of eyestalk, central nervous system and ovary. PLoS ONE 10: e0123848.
6. Christie AE. 2014. Expansion of the *Litopenaeus vannamei* and *Penaeus monodon* peptidomes using transcriptome shotgun assembly sequence data. General and comparative endocrinology 206: 235-254.
7. Bao C, Yang Y, Huang H, Ye H. 2015. Neuropeptides in the cerebral ganglia of the mud crab, *Scylla paramamosain*: transcriptomic analysis and expression profiles during vitellogenesis. Scientific reports 5: 17055.
8. Christie AE. 2016. Expansion of the neuropeptidome of the globally invasive marine crab *Carcinus maenas*. General and comparative endocrinology 235: 150-169.
9. Veenstra JA. 2016. Similarities between decapod and insect neuropeptidomes. PeerJ 4: e2043.
