# Supplementary material for: Insights Into Sexual Maturation and Reproduction in the Norway Lobster (Nephrops norvegicus) via in silico Prediction and Characterization of Neuropeptides and G Protein-coupled Receptors
Source: Front Endocrinol (Lausanne). 2018 Jul 27;9:430. doi: 10.3389/fendo.2018.00430 (PMC6073857; doi:10.3389/fendo.2018.00430)
Supplement: Supplementary Material S5 — Schematics of all detected neuropeptides. [file Data_Sheet_5.pdf]

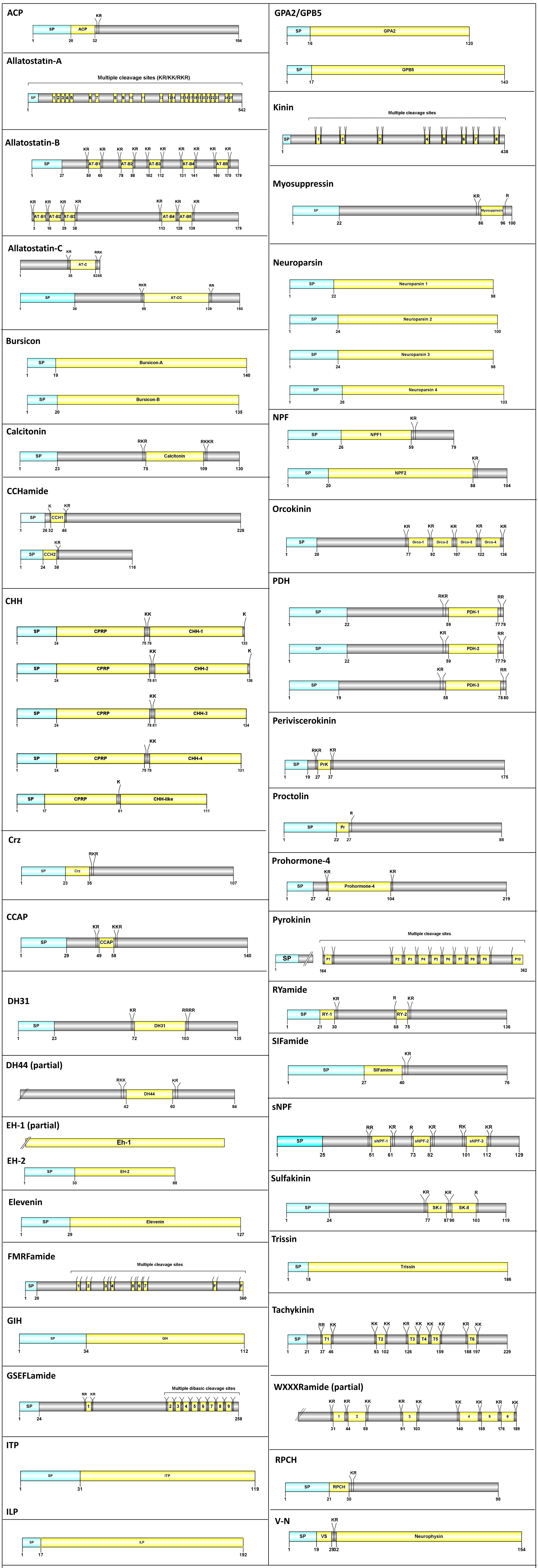

Legend : Schematic diagrams show organisation of neuropeptide precursors, including signal peptide (SP), the mature peptide (yellow) and putative cleavage sites
