# Supplementary material for: Insights Into Sexual Maturation and Reproduction in the Norway Lobster (Nephrops norvegicus) via in silico Prediction and Characterization of Neuropeptides and G Protein-coupled Receptors
Source: Front Endocrinol (Lausanne). 2018 Jul 27;9:430. doi: 10.3389/fendo.2018.00430 (PMC6073857; doi:10.3389/fendo.2018.00430)
Supplement: Supplementary Material S6 — Amino acid sequences of all detected neuropeptides. [file Data_Sheet_6.docx]

**Legends**

Yellow highlight: Signal peptide

Blue highlight: Mature peptide

Red underline: Putative cleavage site(s)

>ACP

MIGWQVMLAVMCLALAPTLAQITFSRSWVPQGKRSGGSTGPLVTPGGGSDLGADPCKDVRLATLTQVASHLADLIDDTFDLSQDDAALALRLKHGLVARRRRMS

>Allatostatin-A

MVGEHGGLGACVLVVAVLLLTTTTTTAHDYLEDLDDPDTSRLLDVLQYYDTEPSYLYDYGKRHSNYGFGLGKRTPVYAFGLGKREGLYSLGLGKRSDLYSFGLGKKSGSYNFGLGKRSVGDHLLSPEVSKVEDDSSPRTKRDVSITEATLEDKRAKYSFGLGKREGSKNKRSMLYGFGLGKRDSGDERREDDDMENRTQQYSFGLDKQDPDMEIEKRPRNYAFGLGKRESDEDSDKRSQMYSFGLGKRDPDMDMEKRPRDYAFGLGKRASSDEDDEERNYAYELGKRPTAYSFGLGKRTFSEADAYDDVNDNDNGDDQLELRDLEQYSDDLKRAASYGFGLGKRTDAPDSGFGRRSYDFGLGKRAGRYAFGLGKRTGPYALDLGKRTGPYAFGLGKRTGPYAFGLGKRAGPYAFGLGKKTGHYAFGLGKRAGPYAFGLRKRSGPYAFGLGKRADPYAFGLGKKAGQYSFGLGKRSGPYSFGLGKRSDSDSDQYTLGRRSGVYSFGLGKRAGPYSFGLGKREVSDDDHNEDEQDIGVEEETSS

>Allatostatin-B1

MMTVQQMCRPWALLVVVLVAGATQVSSSSSSSPQQDDPASSPSHIEEKRVGWSSMRGTWGKRPHLEDAQLDAAEVKRTNWNKFHGSWGKRGEELQAAEDKRTNWNKFQGSWGKRADDLADAELQAAEDKRTNWNKFQGSWGKRADDLADAELQAAEDKRTNWNKFQGSWGKRADDLADA

>Allatostatin-B2_(partial)

KRTNWNKFQGSWGKRNNWRSLQGSWGKRAWNKLQGAWGKRSEDDNGDDLYDETNLEEDLAGNEEQVSPLVLARLMAAAPQKRGWTLWGKRPDNTRVSPRSTNWSSLRGTWGKRSADWNKLRGAWGKRGTDWGQFRGSWGKRTPDMMSVAAPNQA

>Allatostatin-C_(partial)

QQQQQQQQQQQGEEEVKRKRMFVPLSGLPGELPTIKRQIRYHQCYFNPISCFRRK

>Allatostatin-cc

MFVRVGTSGTATTTATAHTSLPLLLPLLLLLLVASAAARVPQQAPRPQYLEVVRPVLPNTALEPLGQLQDAPQQIAETVSTPRKRAAIVLDKLMFALQKALDDSPAASPGQPAPYSRPRTYAAGPMDLQRRGNGDGRLYWRCYFNAVSCF

>Bursicon-A

MGGMSWVLMVLGVATVVWSDECSLTPVIHILSYPGCVSKPIPSFACQGRCTSYVQVSGSKLWQTERSCMCCQESGEREASVVLNCPKVRKGEPTRRKILTRAPIDCMCRPCTDVEEGTVLAQEIANFIHDSPMGNVPFLK

>Bursicon-B

MWITVVLAVVCVVAVPCTHARRYDLECETLPSTIHVAKEEFDEAGRVERTCEEDLAVNKCEGACVSKVQPSVNTPSGFLKDCRCCRETHLRAREVTLTHCYDADGNRLTGDRGTLVIKLREPADCQCFKCGDSTR

>Calcitonin related peptide

MGSSCYLLSAWLVLMGVITLATPHPHPIQESDSGEIPQRLRELLLIRRLISTLNAAESDDVALPYGQQQPMMRKRTCYLNAGLSHGCDYKDLVGATAEKNYWDSLSSPGRKKRSVPEPRLASLQLSHQMQ

>Calcitonin-like diuretic hormone/DH31

MNSTGAVFVSLVVAFIFVSSVNSAAFNREARAVVQIEDPDYVLELLTRLGHSIIRANELEKFVRSSGSAKRGLDLGLGRGFSGSQAAKHLMGLAAANFAGGPGRRRRSSDDGLDLHHDDNLYAQDQAADLAESSR

>CCHamide-1

MTRPRSSTVLLLFFPLVLLCSPPASAHRVLKGGCLNYGHSCLGAHGKRAYVPVHPPVAPRPLLDVLLDALNTPTRSSHYSHARAANSVMGPRASYPEGRVQSPPTSDQLSDMGLDLRGEGYVSGTNDDLESVGAIGSVRGSLDDARDLAQDNVLYYGALDDDYRDARYKRSAVSLPSRGRFGTSPPLGVANNAAPQDRPHILREEHTGKDKMDPKYLDLASFPNWLRR

>CCHamide-2

MSRGMINVFLVVLGVAALSSRAWGSCSQYGHSCFGAHGKRDGDQYARQEPSPLYPEANQLPEFEQRQEDRLSVDEAVTDREIVANARNWLAVLSHRLRQRTSPQSSPSAQSLGYFQ

>CCRFamide

MVSRVGVVVVVMGAWLAGGGQVLANYSQCDVQALRCDVICAFPDLGLHCSRCIRRRPMRFGKRSEEPARTLSEAATGSPLVAIISHPGPTHLQKTKSQDPGQSGLASTTVQYKRTSRVHDAARPVVSSSLRQLINKVLWNEYTARKTLTSGDREQEAHSSQLGRYVSDLQSERGPDYDDSTEGVEYVSFRRSRRSPHHTLDNLGSGGVQSDGRFPVERRSKRSGVHEVIEGLMDLLGLDELPVDPTLYGCHELLLD

>Corazonin

MGMVVVVVMVVVVVVFAVTLAAAQTFQYSRGWTNGRKRSDPNVGVTELLADPPHHLSAHSHPHPPTHTLPKNIEERLRALEAGLNAVLKANSVNFSPGGDEEYYAEN

>CFSH

MLQRLVVQLLVAAVYVSFSVASASEVAGPDSFTEDGRERQEDVSPWVLPQWWWLSSVLSLPQDQDQAHATTSKTNQYLVNAVEEEEDSTLSLVLPAEGNGENDKVRDDMSRAGSSSVTRVGKRSRPCRSQGKNRCRRGLVSLVPASEVHKSWKNDYLSVPEALVQFSQEQTEETVCKDLSVQLFRVDLTEVHLEPYWVRQTSHLGMCPSKLQTRKLGDNVWPNSVVETKCMCQRELCSNIGGDFRCQAVRRPIRTWVRHMDQFMPTEEMVSVGCVCAQKTSPEGNYARIELHS

>CCAP

MTNVSWCGRVGILGVTTVLLLVVLAAHAQAGPVAKRDIGDLLEGKDKRPFCNAFTGCGKKRSDPSMEGLASTSELDALAKHVLAEAKLWEQLQSKMEMMRSYASRMENHPVYRRKRSTPHTQPRQHLTSTPQQKVETEKQ

>CHH-1

MFACRTLCLVVVMVAALWTSGVGGRSVEGASRMEKLLSSISPSSTPLGFLSQEHSVNKRQVFDQACKGVYDRNLFKKLDRVCEDCYNLYRKPFVATTCRENCYSNRVFRQCLDDLLLIDVIDEYVSSVQMVGK

>CHH-2

MMACRTLCLVVVMVAALWTSGVGGRSVEGASRMEKLLSSSNSPSSTPLGFLSQSQEHSVNKRQVFDQACKGVYDRNLFKKLDRVCEDCYNLYRKPFVATTCRENCYSNRVFRQCLDDLLLIDVIDEYVSSVQMVGK

>CHH-3

MMACRTLCLVVVMVAALWTSGVGGRSVEGASRMEKLLSSSNSPSSTPLGFLSQSQEHSVNKRQVFDQACKGVYDRNLFKKLDRVCEDCYNLYRKPFVATTCRQNCFVHDTFPRCVMDLGLDLELFLEFRDMIKG

>CHH-4

MFACRTLCLVVVMVAALWTSGVGGRSVEGASRMEKLLSSISPSSTPLGFLSQEHSVNKRQVFDQACKGVYDRNLFKKLDRVCEDCYNLYRKPFVATTCRQNCFVHDTFPRCVMDLGLDLELFLEFRDMIKG

>CHH-like

MLVTVIVILVFTSSCCGRSWQIDGDEDLQLSQYHSLNKRAAFDSACKGYYDREFWGKLSRVCWDCENLFRQPGYQDKCSEGCFVTTDFTQCVKALLLNVEEYRELAELVRG

>DH44

MAKRTWPNGFSRRRSSGLSLSIDASMKVLREALYMEIIRKKQRQQRQRAQHNKKLLNSIGKRDVTRQLQQEGLQGVYQRDQRK

>EH-1_(partial)

ILASPRVNNTRSFICPPGIAAPLVKFLLTIQYFSMSFKREVVVVVMTVVVMMALVTLSDAATFTSMCIRNCGQCKEMYGDYFHGQACAESCIMTQGISIPDCNNPATFNRFLKRFN

>EH-2

MVGSRKVVVSVLLVLSVMLMALLLLPSAAAAANKVSVCIKNCAQCKIMYHDHFKGGLCADLCVQSGGKFIPDCGRPQTLIPFFLQRLE

>Elevenin

MAASAFLRVRLTTVVLLTTLACLLAYTNAVDCRKFVFAPVCRGIIAKRMVAEKRSSFRPTADTQWNSQYRAPTETEAENLLLASSYDDVMEPRPQEDMVVVRAGSDVVQVPAYVFGVIERSLQGERK

>FLRFamide

MIVAAWVLLTTLTWCCQAHAAPVPPVVAALDPPTDDLLPAQSQEDDMFALPEKRLLKYFLPASQSWGGDAYPIGQEGTKRGYSDRNYLRFGRSDDNSKRSGRNFLRFGRSDANDFEGEEEIPESPEKRNRNFLRFGRDQNRNFLRFGRSRSPMEFATDLQEDVELPVEEKRGAHKNYLRFGRGNNRNFLRFGRGDRNFLRFGRSVDRQLSSLSCEDCDEEQKAREFTSTPSPTTIQPLARAKRDVSAVLGEDSIESSVLRQINARRIKRAAAQNFYIPMAWASELQPEEDGIDVTSFEEPQVDKRFSHDRNFLRFGKRGGSDDYPSSSSSAESPAPVVVVRPVEYPRYVRAPSKNFLRFG

>GPA2

MVKVWVLLVTCLVASATSFKHAWQTPGCHKVGHTRRISIPECLEFDITTNACRGFCESWSVPSAWQTLASNPHQVVTSIGQCCNIMETEDVKVKVMCIQGPRELVFKSASTCDCFHCKKY

>GPB5

MVAVLAAVVVLLVPARAINPQSTLECHRRQYTYKVHKTDDEGRICWDLINVMSCWGRCDSNEIADWKFPYKRSHHPVCMHEETQLTVVTLQHCEDNAAPGTETYSYHEATRCACSVCKTSEASCEGLRYRGARRAPRAEVPRG

> Gonad inhibiting hormone (GIH)

MVTRVASGFSVQRVWLLLVIVVVLCGSVTQQASAWFTNDECPGVMGNRDLYEKVAWVCNDCANIFRINDVGVKCKKDCFHNMDFLWCVYATERHGEIDQFRKWISILRAGRK

> GSEFLamide partial – lack N-terminus

MVRGWPCVVLSWVLLCCWCVLSAALPTHLPDEFDDPVVKRLAGTPHESMIRYFLMAMSNPAGRYKSPQLLNRGVRRIGSEFLGKRSVGKLSDADNPRDFESENCSDDDGTEEENLKKEQFSFTGQYDYDESAGENFGSQEDLFNTKPKRNTRSFHGGVDHDGLKNFFTMLMSKKMGSEFLGKRMGSEFLGKRAMGSEFLGKRAMGSEFLGKRAMGSEFLGKRAMGSEFLGKRALGSEFLGKRAMGSEFLGKRAMGSEF

>Insulin like peptide/Relaxin –

MVVVIAAILVVVSTSWALEPDLIRQIGSRTESEWEVLWNKERLALCRTRLRHNLEAICVKDVYRRSLTSPNHHHIKRSTDICLKVHDSDGEGDIRDKGAVSVNLPTATIEITPSSPDTGQHNIYTRSPFLSVQQANLFVTTWVGGRRGGHYRRRRQSSSITAECCTTVGCTWEEYAEYCPTSSRLRPGVTPI

>Ion_transport_protein

MLVFQGSAVGRACVWLLLLAGLVCPNHQVAGYFYKIRSGTQKEFELINCKQFNKTYYTELSRVCDDCQNIYRKYYNVGVDCKKDCFDNEWFPKCVTYLEHDHLLEEYKKMKEYLNLRDL

>Kinin

MVTVGRWVGLWVRLAVALSSWTASVSLVTSEVMDASPLALPHGRHPNLCTSDQVPSHPIVRCEVGKRQAFHPWGGKRSSFKTAPGLPLSLRQVYLALFQNARPRPPPPSEGELKRASFNPWGGKRSDPLLPASLHEPNAERNTFAPWGGERTAGYFTRDADPPIIEEDPIPYVDVQSDDGEAEDVVKRESFSAWGGKRGSFPADEGKDDWEEEEPTDLFVLDGSLSYPPVDRLRYKRETETYANTPVNSEDSAVKTEAENVKQDAKYDRPTEASGTTVAKRTRFSAWAGKRPDLRVIEDVARKMAEHEPKTPERRAFGAWAGKRSSDVILQDGEDDGPVSAWIGRRLQDTDDKRTFRAWAGKRLSGDKLDDHFLDKKTRFSPWAGKRAEGTLSRLSGSTLKAVLDENSPEDSVDNHKRPSFSAWAGKRSETN

>Myosuppressin

MVFRSSSWSCLLVVGVVVVMGVCVGVGETMPPPICLSQQVPLSPFAKKLCSALINISEFSRAMEEYLGAQAIERSMPVNEPEVKRQDLDHVFLRFGRSQQ

>Neuroparsin-1

MRSDILFTIVIVSLFFFNISEAAPSCDGHGTGAEPTHCDYGSFQDWCGNHVCAKGPGQRCGGEWWENDDCGHGMYCANCGKCAGCTVGIQCWFCDSSS

>Neuroparsin-2

MKCSGISGVVSCSFLLLLIQNAAATPLCPERNEIAPEDLSQCKYGVVLGWCGNAACGKGPDEPCGGRWEENGICGEGMYCVCGYCAGCTSTLECVLGRFC

>Neuroparsin-3

MRSLGFVTSIAVIVVIVIVNETGAAPRCNQGGNSLPANNCKYGIVVDWCGGSVCAKGPGEACGGDWSENGECGAGTYCSCGYCNGCSANLECWFGSYC

>Neuroparsin-4

MKTLTTFITFFVTFFCLVLLFQEAAAAPRCDSHDSPAPTNCKYGTVRDWCRNGVCAKGPGESCGGYWYEYGKCGGGTFCLCGTCIGCSTIDGTCSQSSPAINC

>Neuropeptide_F1

MYRHIWTALMVGVVIVGVSQVSVTQAKPDPNQLAAMADALKYLQELDKYYSQVSRPRFGKRSEYAMVPGDALVSYDGSE

>Neuropeptide_F2

MRGAMMVGAVATVMVAALVAGMASAARPDNSAADTLQAIHEAAMAGILGSAEVQYPNRPSMFKSPVELRQYLDALNAYYAIAGRPRFGKRGNHGAQRTEELYDY

>Periviscerokinin

MRNLVCLRILLLIALIVLGSECRQRKRQDLIPFPRVGKRNHLNDVAGQAGVGVGDTLANWPLLEDPKSNWLAPLMYVQETREQDTEEFTESSNVLPPVWKLLGHKSSDGYRQPIPHINTHTTESHSTEPRHKIKSRQQRSHSPLQLSTFNYLLLRRLLQQVKTQTPAEIETYPQD

>Phoenixin

MTILRGWRYGLFVGGLVGFISAALYPIVIYPMMNVDNYKNIQAVTRKGINQEDVQPGNMKVWSDPFGRKS

>Pigment_dispersing_hormone-1

MRTAVAVAILVLVAMTAVLTRAQELKYPEREVVADMAAQILRVALGPWDTVATVPRKRNSELINSLLGIPKVMNDAGRR

>Pigment_dispersing_hormone-2

MRNSVAVALLMLVVMTAVLTQAQELKYPEREVVAELAAQILRVIQGPWGPMAAGPHKRNSELINSILGLPKVMNDAGRR

>Pigment_dispersing_hormone-3

MVNVGVSLAALLMLALVTSHPLWVDEQTEGGEGLVPLVFVPLSPHHHQVVREPVPKRNSEILNTLLGSQDLSNMRSAGRR

>Proctolin

MARTGLVVVVALVVLAAALTQARYLPTRADDTRLDEIRELLREMLERTAEGANSRINGSGYDKRFMYKRSVPEEGAGEMVQPALNLPQ

>Prohormone-4

MSRSYTTLGQGSALLLLVLLAASASAYDITRRPGLRHYTKRSAYGYGYTQQDYNPNSRVERDCMAYEPFRCPGGEVCISIQYLCDGAPDCPDGYDENPRLCTAAKRPPVEETASFLQSLLASHGPNYLEKLFGTKARNALKPLGGVEAVAVALSESQTIDDFGDTLHLLRSDVEHLRSVFMAVENGDIGMLKSLGIKDSELGDVKFFLEKLVNTGFLD

>Pyrokinin

MHALTWPITLFVYFIFARCTTETLGLEDEWAGLPQASFAQYPPALDDTSEAQPLSLVYNMYPSVTSSDTVPPKSQELQYNSQDTPKILYYSQRPGKRSVDLYDDEATETSQKQEDDHTKRNKRSVNSQEDPERRMKRQTPQHDNEPTDDNDDSTHSWWWPFVAVRRSIFSPRLGKRGDDITNEELAYDDNLATSEYHLDDHDDEYLPEELTEDVTELSSSEVLSESAAALVGKNSVSFIPRLGKRGDGFAFSPRLGKRGADFAFSPRLGRRSDFVFSSRPGKKSDFAFSPRLGKKADFAFSPRLGKRADFAFSPRLGKKADFAFSPRLGKRDSENSNVESRNTKTQASIPRPGRAYFSPRLG

>Red_pigment_concentrating_hormone

MVRASCALMLVVVVFASCVSAQLNFSPGWGKRAAAASGTDPAVASLHPAPPAVLTAASGATAGDSCGTIPVSAVMHIYRLIRTEAARLVQCQEEEYMG

>Ryamide

MSRTICPTLVMFVALLALTAAQGFYTQRYGKRGDTGEVTVRSGFYANRNGRSSPSQGLPEIKIRSSRFIGGSRYGKRSGPAPAAEPEFTPVMNGEADESDMPATLLVGDSVICLLVDVPDIYRCVSRKSTTDEASN

>sNPF

MGVSVIKCWVGLVCCCLLLSQLTAAVPAALQDYDAVNEVYDWLADHGLERRGPPSLRLRFGKRDMGWQVAQRSMPSLRLRFGKRTVDQAESLYDREVVRKDTSTPRLRLRFGKRDTTYGQEEDLASHEQ

>Orcokinin

MTGEVFSVVLLLTLSIFAAAGPIKAAPARSSPQQDAAAGYTGGATVKRFDAFTTGFGHNKRSSEDLDRLGYGFTKRNFDEIDRSGFGFHKRNFDEIDRSGFGFNKRNFDEIDRSGFGFNKRNFDEIDRSGFGFNKR

>SIFamide

MSVQMRVVVALAVVLVIVAVLTDPVSAGYRKPPFNGSIFGKRAGADPLFEPGKGLASVCQVAVEACAAWFPVPEKK

>Sulfakinin

MRWISWTVAVLVVMAAVMLSGVVSAPARPSSLARVLAPVVRHRLEESHLPPALVEELVQDFEDPELLDFHDAAGKREFDEYGHMRFGKRGGVDYDDYGHLRFGRSLTHSDQHHHDTTVN

>Tachykinin

MVRACTWAVLLGAVVVMGVVAAAGEGQDTPQDRERRAPSGFLGMRGKKDAATALDDNTAASEYSSLPDPYPLYGLRDNNLPMLFAVPWKTKKAPSGFLGMRGKKSDEEVFSDATADNDLEILLKRAPSGFLGMRGKKAPSGFLGMRGKKAPSGFLGMRGKKYYDDDSDMDAYIQALTAVVDGQQQKRAPSGFLGMRGKKAYYSENPDEEISMAGVDKRTPSGFLGMRGS

>Trissin

MNSLAIFFAWALVGGTWAWSSSEVSCTSCGSECQSACGTRNFRACCFNFQRRRRADPRSRSAGVIGADSMALEGLLKPNVSGTKDSHLSQILHLLSRALAESKTDPAFYKDPPSLSSVLSPLVQESTEEETDDNSDLLPSSVGDSDGHSLDNVIYLAFKRPSPSLNQLQHQNFHQRYTPPPTNINK

>Vasopressin-neurophysin

MQLGVAVVVMTVVVGSTTACFITNCPPGGKRSGPTAQLGRTRTCTACGPGLQGRCLGPEICCVLGNGCFLGTREARMCHAENLVPVTCANRDLKSCGRMQEGRCAAAGLCCTEMKCEFDSSCTVEGREESVGKQRAERQHFTFMSSLPEDQWNL

>WXXXRamide_(partial)

SSQQQHRQLPQPRLQVNSGWESNQSLWGKRDGGGPFWIARGKRQETEEGNGGPFWIARGKKDIDSGVYWDTAVQDDDGQWQDDVTYPHKRQDDGGGPFWISRGKKDTPALLSVGHPSLWGNRGGKSEDERTFWVARGKKDTTATVDGRAPFWISRGKKENKGNESELFWISRGKREGETPPFWVSRGKK
