# Supplementary material for: Insights Into Sexual Maturation and Reproduction in the Norway Lobster (Nephrops norvegicus) via in silico Prediction and Characterization of Neuropeptides and G Protein-coupled Receptors
Source: Front Endocrinol (Lausanne). 2018 Jul 27;9:430. doi: 10.3389/fendo.2018.00430 (PMC6073857; doi:10.3389/fendo.2018.00430)

Adipokinetic hormone/corazonin-related peptide (ACP) receptor

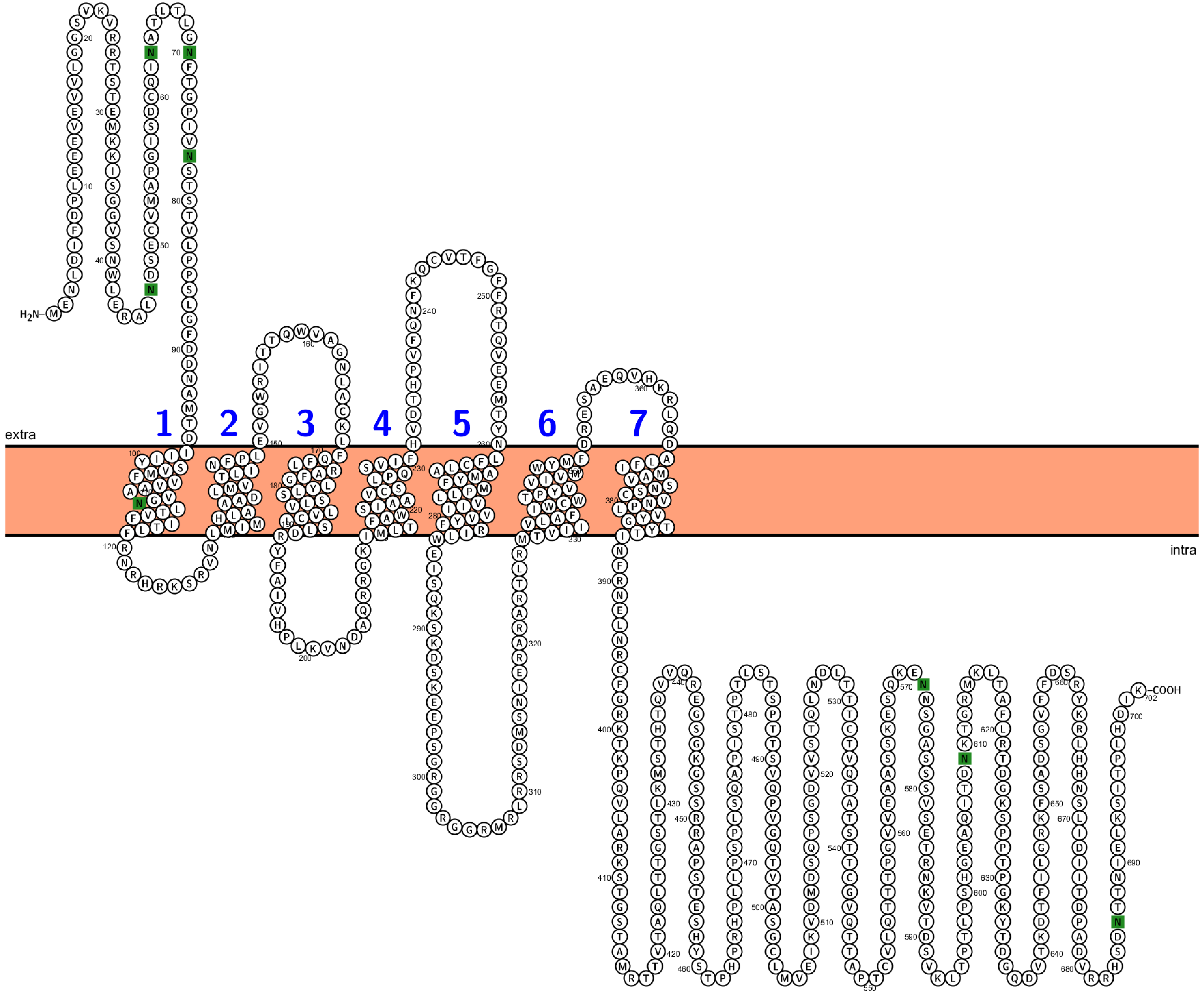

Corazonin (Crz) Receptor

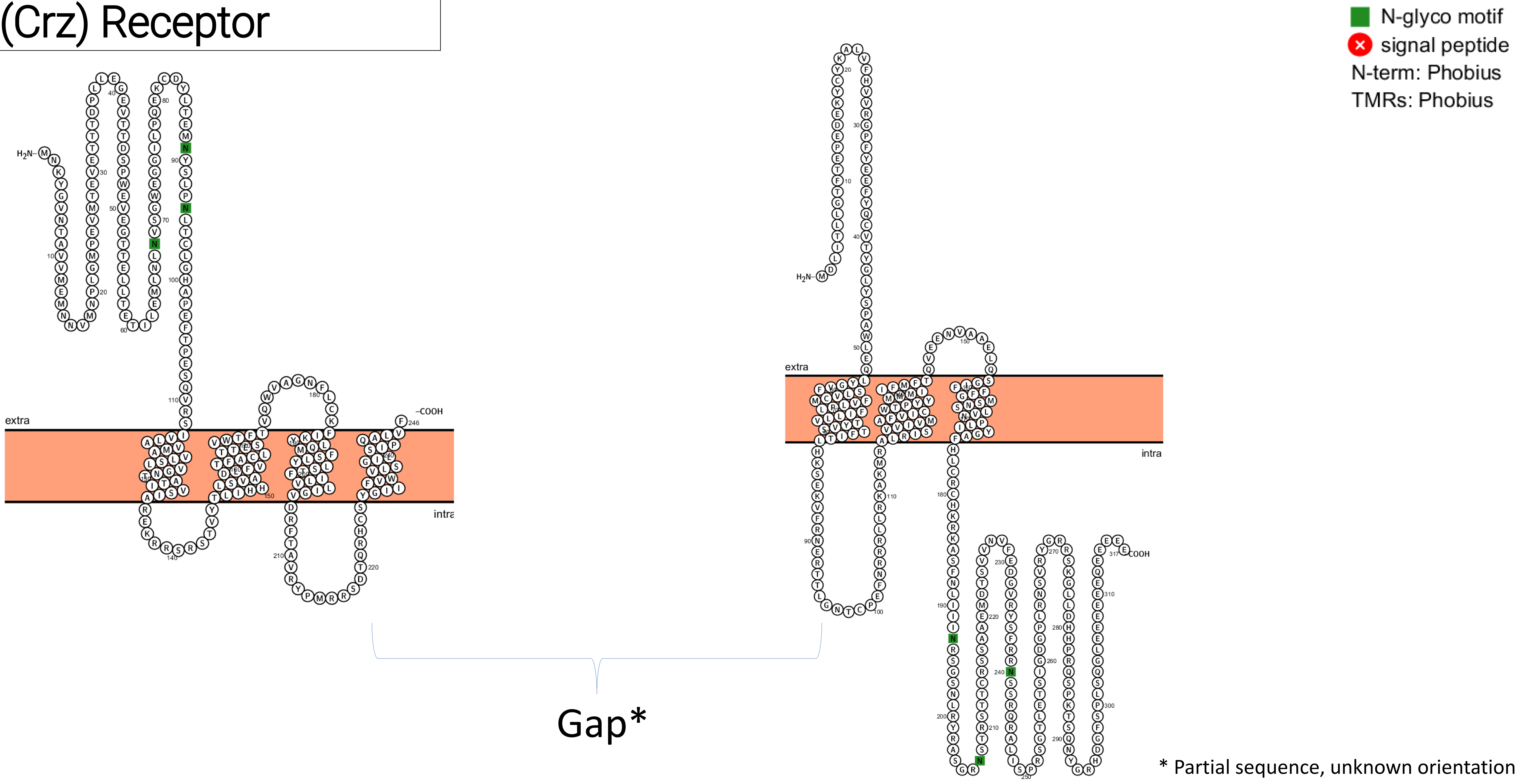

* Partial sequence, unknown orientation

Red pigment concentrating hormone (RPCH) Receptor

N-glyco motif
signal peptide
N-term: Phobius
TMRs: Phobius

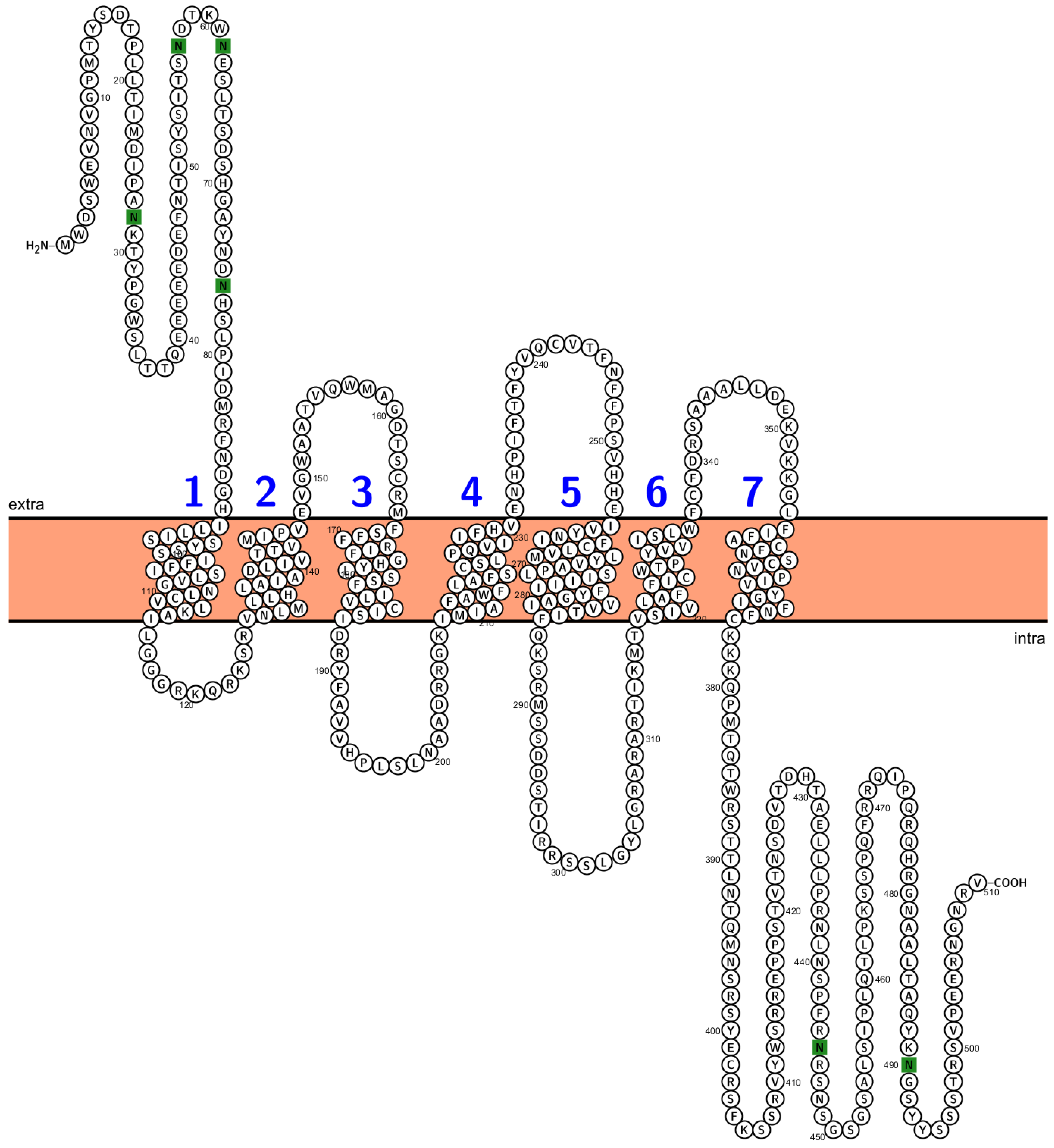

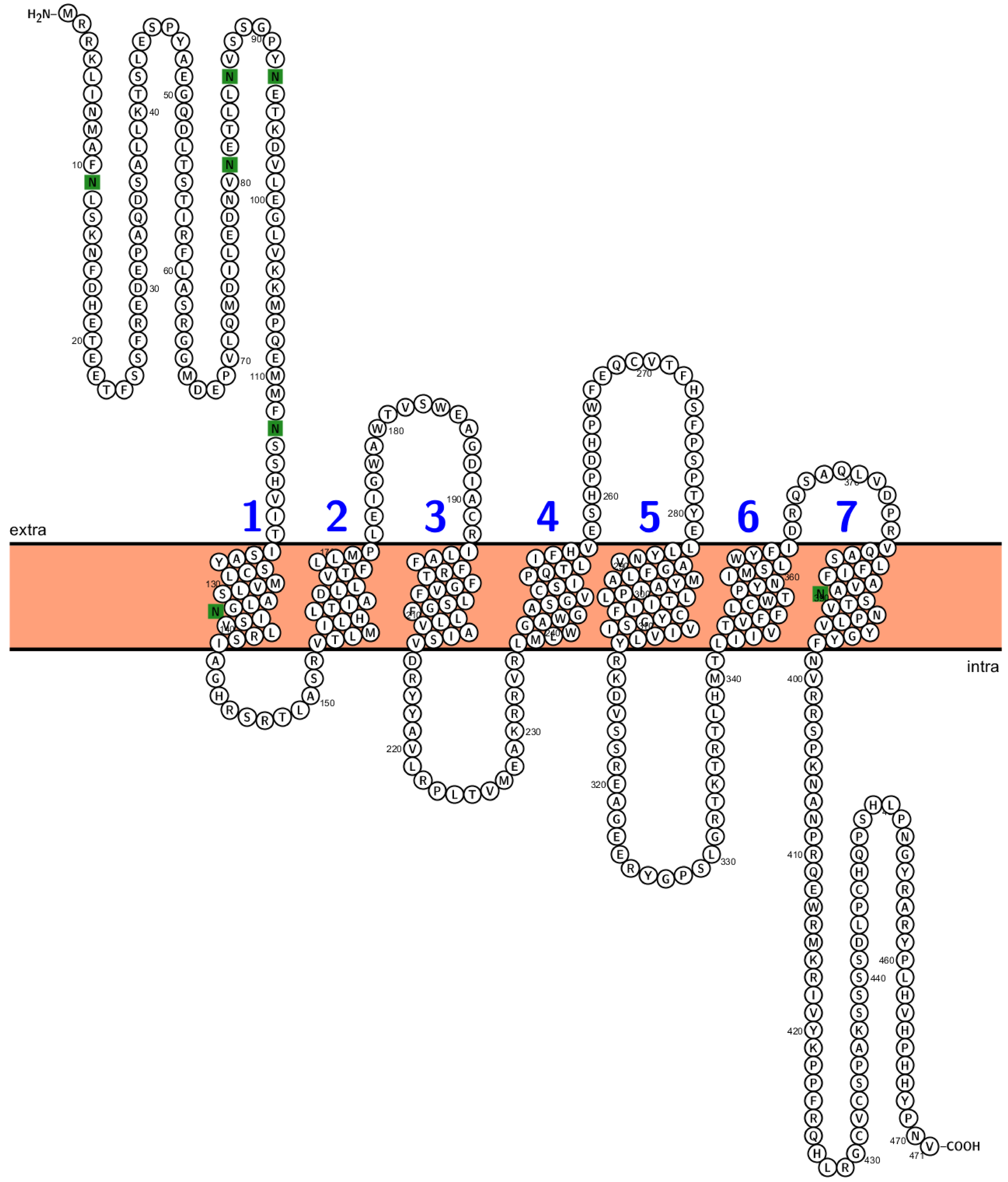

Crustacean cardioactive peptide (CCAP) receptor

N-glyco motif
signal peptide
N-term: Phobius
TMRs: Phobius

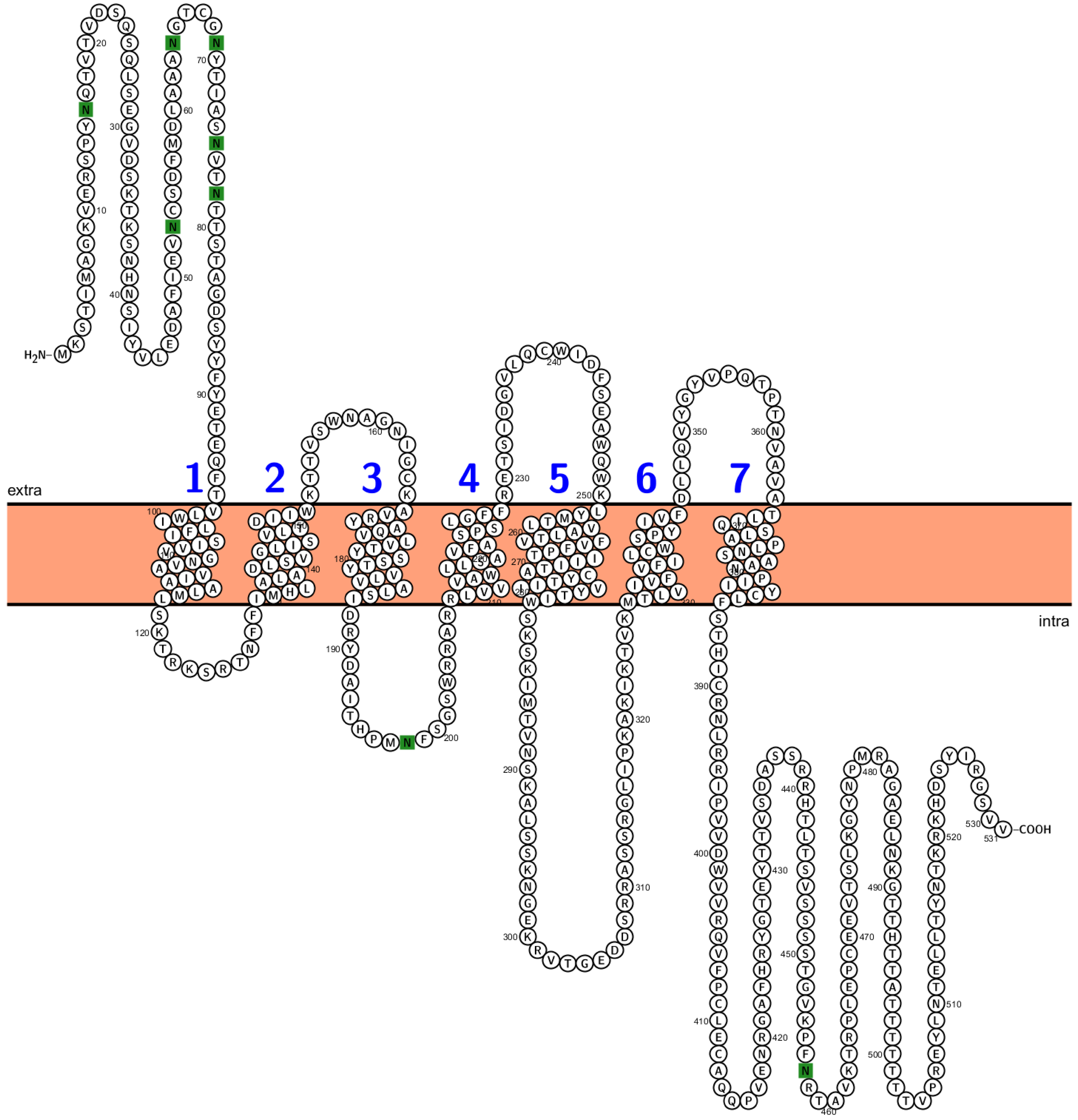

Vasopressin - Neurophysin (V-N) Receptor

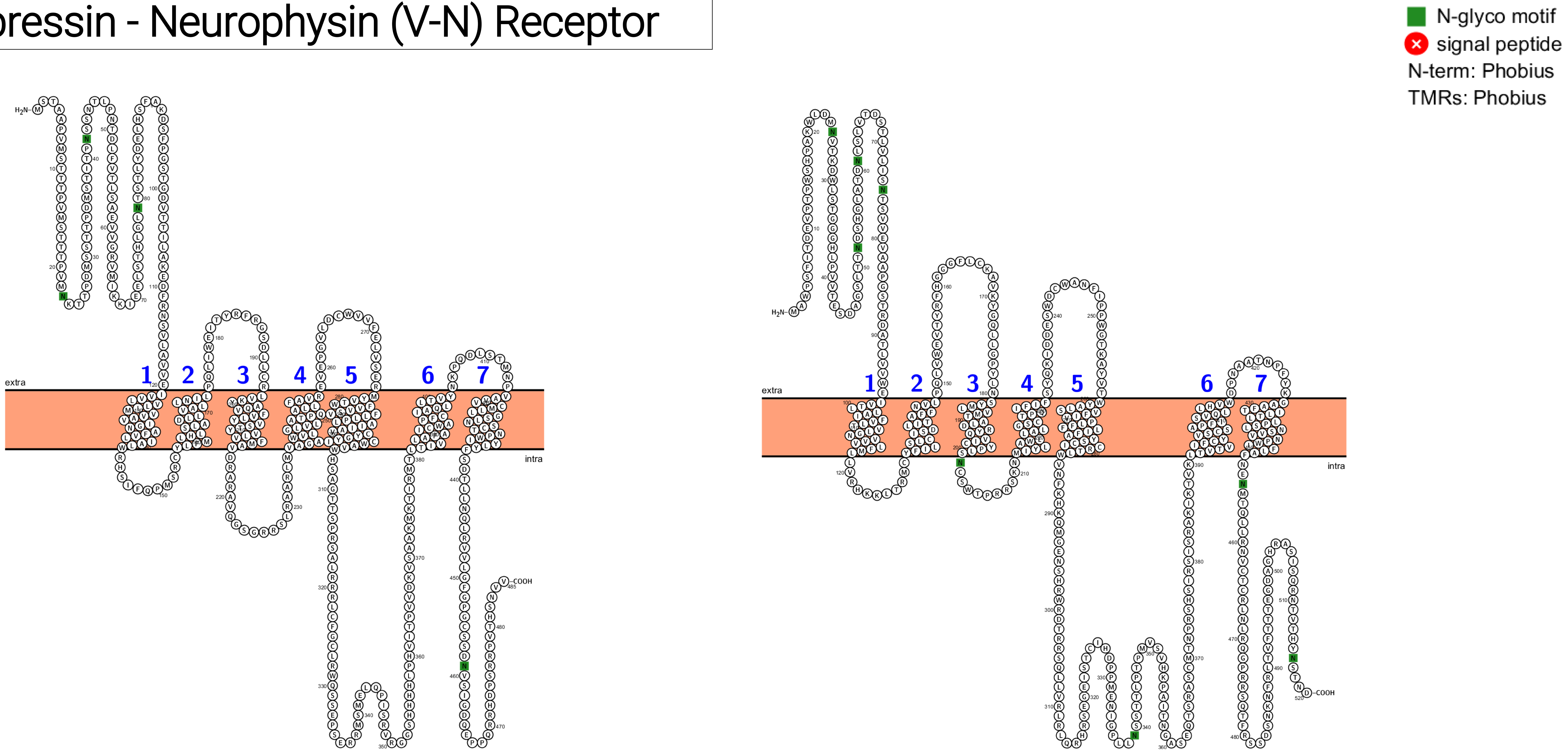

Supplement: Supplementary Material S8 — Illustrations of ACPR, CrzR, RPCHR, CCAPR and V-NR. [file Data_Sheet_8.pdf]
