# Supplementary material for: Insights Into Sexual Maturation and Reproduction in the Norway Lobster (Nephrops norvegicus) via in silico Prediction and Characterization of Neuropeptides and G Protein-coupled Receptors
Source: Front Endocrinol (Lausanne). 2018 Jul 27;9:430. doi: 10.3389/fendo.2018.00430 (PMC6073857; doi:10.3389/fendo.2018.00430)
Supplement: Supplementary Material S9 — Phoenixin sequences used in this study. [file Data_Sheet_9.docx]

**Legends**

Blue highlight: PNX-20 predicted mature peptide

Red underline: Putative cleavage site(s)

>*Nephrops norvegicus* PNX-20

MTILRGWRYGLFVGGLVGFISAALYPIVIYPMMNVDNYKNIQAVTRKGINQEDVQPGNMKVWSDPFGRKS

>*Sagmariasus verreauxi* PNX-20

MALLQGWRYGLLVGGLVGFISAALYPIIIYPIMNTDSYKKIQKETRKGINQEEIQPGNMKVWSDPFGRKPQ

>*Eriocheir sinensis* PNX-20

MMGILQGWRYNLLVGGLIGFIGATLYPIVIYPMMNVDQYKKAQSENRKGINQEEVQPGNMKVWSDPFERKK

>*Scylla paramamosain* PNX-20

MTILRGWRYSLFLGGLIGFIGATLYPIVIYPMMNSSQYKQAQIENRRGINQEEIQPGNMNVWSDPFDRKK

>*Procambarus clarkii* PNX-20

MSKLSGWRYHVFIGGLVGFIGAALYPIIIYPMMNIDAYKKIQAENRKNVNQQEIQPGNMKVWTDPFGRKS

>*Cherax quadricarinatus* PNX-20

MTMVNLHGWRYGMFVGCLVGFISATVYPIIIYPMMNIDTYKKIQAENRKNIKQEEIQPGNMKVWSDPFGRKS

>*Penaeus monodon* PNX-20

MSTLRGWRYGLFLGGLVGFISAALYPIIIYPMMHVDDYKKIQAENRKGVNQEEIQPGNMKVWSDPFDRK

>*Penaeus vannamei* PNX-20

MSTLRGWRYGLFLGGLVGFIGAALYPIIIYPMMHVDDYKKIQAENRKGVNQEEIQPGNMKVWSDPFDRK

>*Macrobrachium rosenbergii* PNX-20

MTILRGWKYGVFIGGIVGFISAALYPIVVYPMMHVDEYKKAQAINRQGINQEEIQPGNMKVWTDPFQRK

> *Homo sapiens* PNX-20

MSRNLRTALIFGGFISLIGAAFYPIYFRPLMRLEEYKKEQAINRAGIVQEDVQPPGLKVWSDPFGRK

> *Mus musculus* PNX-20

MAAARNLRTALIFGGFISMVGAAFYPIYFRPLMRLEEYQKEQAVNRAGIVQEDVQPPGLKVWSDPFGRK
